# A Systematic Review of Safety and Efficacy of Factor XI/XIa Inhibitors in Patients With ESKD on Hemodialysis

**DOI:** 10.1016/j.ekir.2024.10.007

**Published:** 2024-10-15

**Authors:** Daniel Steiner, Daniel Kraemmer, Stephan Nopp, Oliver Königsbrügge, Cihan Ay

**Affiliations:** 1 Division of Hematology and Hemostaseology, Department of Medicine I, Medical University of Vienna, Vienna, Austria

**Keywords:** anticoagulants, embolism and thrombosis, factor XI, hemorrhage, renal dialysis, renal insufficiency

## Abstract

**Introduction:**

Factor XI/XIa (FXI/XIa) has emerged as a potential target for antithrombotic therapy, driven by preclinical evidence showing the role of FXI/XIa inhibition for preventing thrombosis without impeding hemostasis. This is particularly promising for patients at high risk of both thromboembolic events and bleeding, such as patients with end-stage kidney disease (ESKD) on hemodialysis (HD).

**Methods:**

We systematically searched Embase, MEDLINE, and ClinicalTrials.gov for randomized controlled trials evaluating FXI/XIa inhibitors in patients with ESKD on HD, without restricting inclusion to specific comparators or indications. Interventional treatment arms were pooled, and study results were synthesized by fitting random-effects models, calculating odds ratios (ORs) and 95% confidence intervals (CIs).

**Results:**

Five phases 2 studies encompassing 1270 participants were identified, investigating gruticibart, IONIS-FXI_Rx,_ osocimab, or fesomersen in the general HD population and using placebo as a comparator. Four studies were fully published and included in the meta-analysis. Use of FXI/XIa inhibitors was associated with an OR of 0.80 (95% CI = 0.47–1.35) for clinically relevant bleeding, 0.51 (95% CI = 0.21–1.28) for major bleeding, and 0.90 (95% CI = 0.49–1.68) for clinically relevant nonmajor bleeding. The ORs for thromboembolic events and all-cause mortality were 0.66 (95% CI = 0.28–1.56) and 0.46 (95% CI = 0.15–1.40), respectively.

**Conclusion:**

Currently available evidence does not indicate a significantly increased bleeding risk of FXI/XIa inhibitors in patients with ESKD on HD compared to placebo. Their efficacy and their association with all-cause mortality need to be investigated in sufficiently powered, randomized controlled phase 3 trials.

## Graphical abstract

**Figure:**
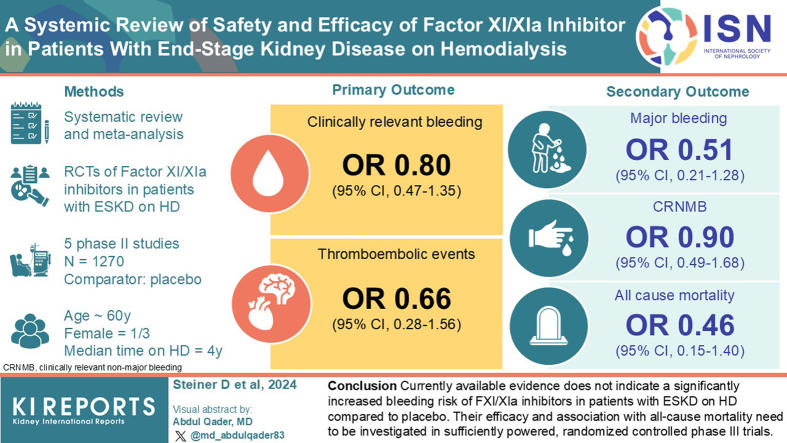

See Commentary on Page 4

Chronic kidney disease is a common condition posing a substantial burden on patients and health care systems.^1^ Patients with chronic kidney disease are at risk for cardiovascular complications, especially for thromboembolic events.^2^^,^^3^ In addition, the risk of bleeding-with and without anticoagulation-is elevated.^4^ The risk of both thromboembolism and bleeding further increases in patients with end-stage kidney disease (ESKD) on hemodialysis (HD), resulting in the clinical challenge of balancing thromboembolic and bleeding risk in this vulnerable patient population.^5^, ^6^, ^7^, ^8^, ^9^ However, patients with chronic kidney disease, particularly those with ESKD, were underrepresented or excluded from most clinical trials investigating antithrombotic or anticoagulant therapies in general, resulting in limited evidence on which to base clinical decisions.^10^^,^^11^ Recently, 3 randomized controlled trials evaluated direct oral anticoagulants versus vitamin K antagonists in patients with atrial fibrillation and ESKD on HD.^12^, ^13^, ^14^ However, these trials struggled with recruitment issues, leading to underpowered results, which did not allow to draw definitive conclusions.^15^ Consequently, none of the available direct oral anticoagulants is licensed for use in patients with ESKD on HD outside of the United States up to now. Overall, it is an ongoing debate whether patients with ESKD on HD might benefit from anticoagulation and, if so, which treatment strategy should be recommended or whether anticoagulation would constitute an intervention with potential net clinical harm in this cohort.^16^

In recent years, anticoagulant drug development strategies have focused on inhibiting the intrinsic pathway, more specifically, factor XI and/or its active form factor Xia, to prevent pathological thrombosis while maintaining physiological hemostasis.^17^, ^18^, ^19^ This theory is supported by epidemiological observations. Higher factor XI levels are associated with an increased risk of thrombosis, and the incidence of thromboembolic events in persons with factor XI deficiency is reduced.^20^, ^21^, ^22^, ^23^, ^24^, ^25^ In addition, the bleeding tendency in persons with factor XI deficiency is relatively mild with rare spontaneous bleedings, and the correlation of factor XI levels and bleeding phenotype is poor.^26^, ^27^, ^28^ Recently, 4 proof-of-concept studies evaluated the use of different FXI/XIa inhibitors after total knee arthroplasty for the prevention of venous thromboembolism compared to low-molecular-weight heparin.^29^, ^30^, ^31^, ^32^ Metaanalyses of these trials not only showed the superiority of FXI/XIa inhibitors in preventing venous thromboembolism, but also a significant reduction in major and clinically relevant nonmajor bleeding events compared to low-molecular-weight heparin.^33^^,^^34^ In a more recent metaanalysis including 4 additional randomized controlled trials, a reduction in bleeding events and trial-defined efficacy end points compared with low-molecular-weight heparin and a trend toward less bleeding events compared with oral factor Xa inhibitors has been suggested.^35^

Given the high risk of thrombosis and the exposure to artificial surfaces in the HD system, inhibiting FXI/XIa emerged as a promising potential therapeutic strategy in patients with ESKD on HD.^36^ This might be beneficial in the general population of patients on HD, including those with and without atrial fibrillation. Furthermore, the high risk of bleeding in these patients warrants safer anticoagulants. Therefore, we conducted a systematic review and metaanalysis of the literature on the safety (i.e., bleeding risk) and efficacy (i.e., prevention of thromboembolic events) of FXI/XIa inhibitors in patients with ESKD on HD in randomized controlled trials.

## Methods

This systematic review is presented according to the Preferred Reporting Items for Systematic Reviews and Meta-Analyses (PRISMA) 2020 statement (Supplementary Table S1).^37^ Prior to study initiation, we registered the protocol in the International prospective register of systematic reviews (PROSPERO; ID: CRD42024536740).

### Eligibility

Studies were selected according to the following criteria: study design (randomized trials, phase 2 and phase 3), population (adult patients with ESKD on HD), and interventions (FXI/XIa inhibitors vs. any comparator). We did not consider observational studies, case series, and case reports. No restrictions regarding indication for anticoagulation, study setting, and language were applied.

### Outcomes

The primary safety outcome was clinically relevant bleeding, that is, the composite of major bleeding and clinically relevant nonmajor bleeding, according to the definition in the identified studies. Secondary safety outcomes included major bleeding and clinically relevant nonmajor bleeding. The primary efficacy outcome was thromboembolic events according to definition in the identified studies. The secondary efficacy outcome was all-cause mortality.

### Information Sources and Search Strategy

We performed a systematic search in Embase, MEDLINE, and ClinicalTrials.gov from inception to date of search (April 17, 2024). In addition, we reviewed references for primary studies and review articles. The search strategy was based on predefined search terms using subject headings and free-text formulations with regard to population, intervention, and study design. The search terms related to study design were selected according to the strategy suggested in the Cochrane Handbook for Systematic Reviews of Interventions.^38^ The exact search strategies for each database are provided in Supplementary Table S2.

### Study Selection, Data Extraction, and Risk of Bias Assessment

Search results were screened independently for eligibility by 2 authors (DS and SN). Full texts of eligible records were then examined for inclusion. Next, extraction of study and participant characteristics as well as outcomes was performed independently by the same 2 authors. Risk of bias was assessed independently by 2 authors (DS and DK) with the revised Cochrane risk of bias tool (ROB 2).^39^ Disagreement at any of the described steps was resolved by discussion and consensus. In the case of persisting disagreement, a third author (CA) was consulted.

### Statistical Analysis

We pooled treatment arms of FXI/XIa inhibitors and compared the pooled results to placebo and/or active comparator, whichever was applicable. Because we expected outcomes to be reported as absolute frequencies and proportions, we calculated ORs with 95% CIs for primary and secondary outcomes by synthesizing individual study results using random-effects models. Between-study heterogeneity was estimated with the restricted maximum likelihood estimator and quantified using the Q-test for heterogeneity and the I^2^ statistic.^40^ In addition, prediction intervals for primary and secondary outcomes are provided. Because there were zero events for some outcomes, we used a continuity correction of 0.5.

Due to the small number of included studies, we were not able to assess potential publication bias. For the same reason, we did not perform subgroup analyses by type of FXI/XIa inhibitor. We performed several sensitivity analyses. First, we excluded results from 1 study, which was deemed to be at high risk of bias. In addition, we performed an analysis including an unpublished study with results available on ClinicalTrials.gov. Lastly, we performed an analysis of the highest dosing group of FXI/XIa inhibitors per study compared to placebo. All analyses were done in R using the PRISMA2020 and metafor packages.^41^, ^42^, ^43^

## Results

**Figure 1:**
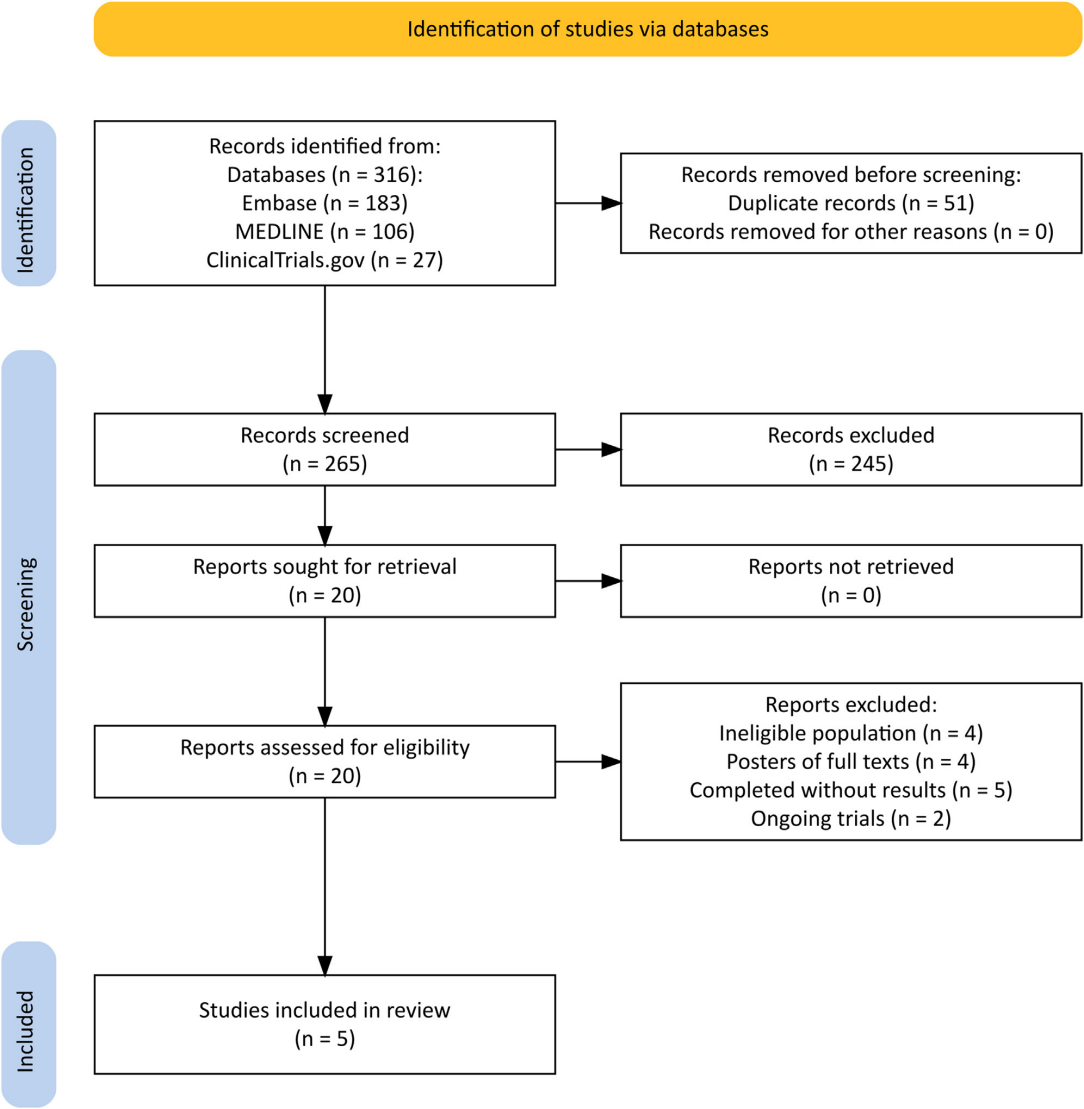
PRISMA flow diagram. The systematic search of databases (Embase, MEDLINE, and ClinicalTrials.gov) was conducted on April 17, 2024.

The literature search was conducted on April 17, 2024, and 316 records were identified. After exclusion of duplicates and screening of titles and abstracts, we assessed 20 reports for eligibility. Of those, 5 studies fitted our inclusion criteria, encompassing 4 fully published reports^44^, ^45^, ^46^, ^47^ and 1 completed study with results posted on ClinicalTrials.gov, the EMERALD study^48^ (Figure 1). Further, we identified 3 completed phase 1 studies without published results and 1 ongoing phase 2 study on ClinicalTrials.gov (Supplementary Table S3). We did not identify any completed or ongoing phase 3 study.

The FXI/XIa inhibitors investigated in the included studies were monoclonal antibodies (gruticibart and osocimab) and antisense oligonucleotides (IONIS-FXI_Rx_ and fesomersen), all of which were compared to placebo (Table 1). All studies included patients from the general HD population for the prevention of thromboembolic events, without specifically targeting patients with atrial fibrillation. Detailed inclusion and exclusion criteria are available in Supplementary Table S4. Overall, 1270 participants were randomized, and all studies had at least 2 dosing arms for the intervention. Detailed characteristics of included studies are shown in Table 1.

**Table 1:** Characteristics of included studies

Characteristics	Lorentz *et al.*^44^	CS4^45^	CONVERT^46^	RE-THINC^47^	EMERALD^48^
Study design	Single-center, national, randomized, controlled phase 2 trial	Multicenter, national, randomized, controlled phase 2 trial	Multicenter, international, randomized, controlled phase 2b trial	Multicenter, international, randomized, controlled phase 2b trial	Multicenter, international, randomized, controlled phase 2 trial
Participants	*N* = 24 18–80 yrs, HD >3 mo 3 times/wk for a minimum of 3 h per session; United States	*N* = 43 18–80 yrs, HD >3 mo 3 times/wk for a minimum of 3 h per session; Canada	*N* = 686 18 yrs or older, HD >3 mo 3 times/wk for a minimum of 3 h per session, ≥50 kg; North America, Europe, Asia, Australia	*N* = 307 18 yrs or older, HD >3 mo 3 times/wk for a minimum of 3 h per session; North America, Europe, Asia	*N* = 210 18–85 yrs, HD >3 mo 3 times/wk for a minimum of 3 h per session; Canada, Europe, Asia
Intervention	Gruticibart^a^	IONIS-FXI_Rx_	Osocimab	Fesomersen^b^	IONIS-FXI_Rx_
Drug type	Monoclonal antibody	Antisense oligonucleotide	Monoclonal antibody	Antisense oligonucleotide	Antisense oligonucleotide
Mechanism	Binds factor XI, inhibits activation by factor XIIa	Inhibits factor XI messenger RNA	Binds and inhibits factor XIa	Inhibits factor XI messenger RNA	Inhibits factor XI messenger RNA
Route	Into dialysis line	Subcutaneous	Subcutaneous	Subcutaneous	Subcutaneous
Interval	Single dose	D 1, 5, 8, 12, and 15, then wk	Mo	Mo	Wk
Dosing	0.25 and 0.50 mg/kg	200 and 300 mg	105 mg loading + 52.5 mg maintenance, 210 mg loading + 105 mg maintenance	40, 80, and 120 mg	200, 250, and 300 mg
Control	Placebo	Placebo	Placebo	Placebo	Placebo
Primary outcome	Adverse events	Adverse events	Clinically relevant bleeding, adverse events	Clinically relevant bleeding	Clinically relevant bleeding
Follow-up	21 d^c^	162 d	Up to 22 mo (6 mo main treatment, 12 mo extension treatment)	Up to 16 mo (12 mo treatment)	260 d
Year of publication	2021	2022	2024	2024	-
Funding	National Institutes of Health, Oregon Health & Science University	Ionis Pharmaceuticals Inc.	Bayer AG	Bayer AG	Ionis Pharmaceuticals Inc.
Notes	Patients received heparin-free dialysis sessions	2-part study: open-label pharmacokinetics cohort, followed by randomized controlled trial	-	-	Completed 2019, results on ClinicalTrials, no publication available

HD, hemodialysis.

a Gruticibart is formerly known as AB023/xissomab 3G3.

b Fesomersen is a modified version of IONIS-FXIRx, sharing the same sequence but containing an N-acetyl galactosamine conjugation to direct it to hepatocytes, the cells where FXI is expressed.^49^

c One bleeding episode after 32 days was reported.

Baseline clinical characteristics of randomized patients were generally well-balanced between studies, with an age of about 60 years, approximately one-third were women, and a median time on dialysis of about 4 years (Table 2 and Supplementary Table S5). One study excluded patients on antiplatelet therapy and performed heparin-free dialysis sessions.^44^ The 2 largest studies reported rates of patients with atrial fibrillation, which were approximately 5%.^46^^,^^47^ In the CONVERT trial, the included patients with atrial fibrillation were reportedly not considered to be candidates for anticoagulation by their treating physician, without further specific reason provided.^46^ In the study by Lorentz *et al.*,^44^ patients underwent heparin-free dialysis, whereas the vast majority of patients in the other studies received HD system anticoagulation (Table 2 and Supplementary Table S5).

**Table 2:** Baseline characteristics of randomized patients

Study	Lorentz *et al.*^44^			CS4^45^			CONVERT^46^			RE-THINC^47^				EMERALD^48^			
Dose	0.25 mg/kg (*n* = 8)	0.50 mg/kg (*n* = 8)	Placebo (*n* = 8)	200 mg (*n* = 15)	300 mg (*n* = 15)	Placebo (*n* = 13)	105 mg, 52.5 mg (*n* = 232)	210 mg, 105 mg (*n* = 224)	Placebo (*n* = 230)	40 mg (*n* = 77)	80 mg (*n* = 79)	120 mg (*n* = 76)	Placebo (*n* = 75)	200 mg (*n* = 53)	250 mg (*n* = 54)	300 mg (*n* = 50)	Placebo (*n* = 53)
Age, yr, mean (SD)	55.8 (7.6)	53.4 (5.0)	52.5 (9.2)	-	-	-	-	-	-	60.4 (13.2)	58.0 (14.4)	57.7 (13.3)	58.6 (11.9)	61 (14)	63 (12)	58 (14)	61 (13)
Age, yr, median (range)	-	-	-	56 (31–77)	58 (29–76)	63 (40–80)	61 (28–91)	61 (25–90)	60 (24–90)	-	-	-	-	-	-	-	-
Women, *n* (%)	1 (12.5)	3 (37.5)	1 (12.5)	5 (33.3)	12 (80.0)	4 (30.8)	89 (38.4)	81 (36.2)	80 (34.8)	35 (45.5)	20 (25.3)	30 (39.5)	22 (29.3)	23 (43.4)	19 (35.2)	20 (40.0)	19 (35.8)
BMI, kg/m^2^, mean (SD)	27.8 (3.9)	27.9 (5.4)	24.7 (2.9)	nr	nr	nr	-	-	-	-	-	-	-	nr	nr	nr	nr
BMI >30 kg/m^2^, *n* (%)	-	-	-	nr	nr	nr	74 (31.9)	65 (29.0)	62 (27.0)	19 (24.7)	20 (25.3)	27 (35.5)	18 (24.0)	nr	nr	nr	nr
Etiology of kidney disease, *n* (%)	-	-	-	-	-	-	-	-	-	-	-	-	-	-	-	-	-
Hypertension	6 (75)	5 (62.5)	4 (50)	0 (0)	1 (6.7)	2 (15.4)	51 (22.0)	67 (30.0)	50 (21.7)	14 (18.2)	14 (17.7)	19 (25.0)	15 (20.0)	nr	nr	nr	nr
Diabetes	0	2 (25)	0 (0)	5 (33.3)	8 (53.3)	5 (38.5)	65 (28.0)	53 (23.6)	59 (25.6)	14 (18.2)	21 (26.6)	19 (25.0)	19 (25.3)	nr	nr	nr	nr
Glomerulo-nephritis	nr	nr	nr	4 (26.7)	2 (13.3)	2 (15.4)	32 (13.8)	30 (13.4)	33 (14.3)	13 (16.9)	14 (17.7)	16 (21.1)	9 (12.0)	nr	nr	nr	nr
Polycystic kidney disease	1 (12.5)	0 (0)	0 (0)	2 (13.3)	0 (0)	0 (0)	23 (9.9)	23 (10.3)	21 (9.1)	10 (13.0)	11 (13.9)	1 (1.3)	8 (10.7)	nr	nr	nr	nr
Pyelonephritis	nr	nr	nr	nr	nr	nr	11 (4.7)	10 (4.5)	10 (4.3)	nr	nr	nr	nr	nr	nr	nr	nr
Others/multiple	1 (12.5)	1 (12.5)	4 (50)	4 (26.7)	4 (26.7)	4 (30.8)	50 (21.5)	41 (18.3)	57 (24.8)	26 (33.8)	19 (24.1)	21 (27.6)	24 (32.0)	nr	nr	nr	nr
Time on dialysis, yr, mean (SD)	9.6 (6.7)	7.6 (4.9)	8 (5.3)	nr	nr	nr	-	-	-					nr	nr	nr	nr
Time on dialysis, yr, median (IQR)	-	-	-	nr	nr	nr	4.05 (2.0–7.2)	4.0 (2.0–7.5)	3.85 (1.8–7.0)	3.7 (1.9–6.3)	4.3 (1.7–7.3)	4.2 (2.0–7.5)	3.0 (1.1–6.9)	nr	nr	nr	nr
HD system anticoagulation, *n* (%)	0^a^	0^a^	0^a^	12 (80.0)	14 (93.3)	11 (84.6)	221 (95.3)	218 (97.3)	218 (94.8)	75 (97.4)	73 (92.4)	74 (97.4)	74 (98.7)	nr	nr	nr	nr
Low dose aspirin, *n* (%)	0^a^	0^a^	0^a^	5 (33.3)	7 (46.7)	6 (46.2)	97 (41.8)	95 (42.4)	98 (42.6)	36 (46.8)	38 (48.1)	35 (46.1)	33 (44.0)	nr	nr	nr	nr

BMI, body mass index; HD, hemodialysis; IQR, interquartile range; nr, not reported.

a In the study by Lorentz *et al*.^44^, patients with antiplatelet therapy were excluded and all included patients received heparin-free dialysis sessions.

### Risk of Bias

We judged the study by Lorentz *et al.*^44^ to be at high risk of bias based on measurement of the outcome. Therefore, we conducted a sensitivity analysis excluding this study. Further, we deemed 3 of the included studies to be at some risk of bias based on application of naïve per-protocol analyses and/or measurement of the outcome.^45^^,^^46^^,^^48^ Because the results of the EMERALD study were only published on ClinicalTrials.gov, limiting the available information, we only included the 4 fully published reports in our meta-analysis. We then conducted a sensitivity analysis that included data from the EMERALD study. The detailed risk of bias assessment is presented in Supplementary Table S6.

### Primary Outcomes

All included studies reported clinically relevant bleeding episodes, whereas only 2 fully published reports and the EMERALD study reported thromboembolic events. The definition of clinically relevant bleeding and thromboembolic events per study is outlined in Supplementary Table S7.

**Figure 2:**
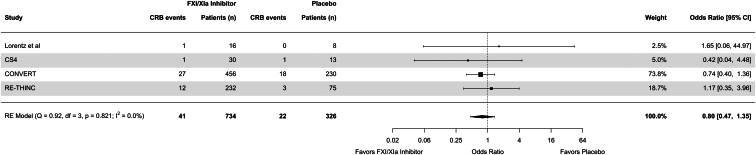
Forest plot for clinically relevant bleeding events. CI, confidence interval; CRB, clinically relevant bleeding; RE, random effects.

**Figure 3:**
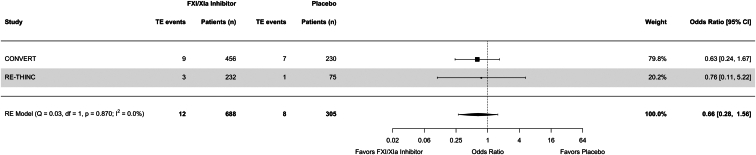
Forest plot for thromboembolic events. CI, confidence interval; RE, random effects; TE, thromboembolic.

Use of FXI/XIa inhibitors was associated with an OR of 0.80 (95% CI = 0.47–1.35) for clinically relevant bleeding compared to placebo (Figure 2). Notably, in the study by Lorentz *et al.*,^44^ 1 bleeding event in the intervention arm was reported to have occurred after 32 days even though the follow-up time was limited to 21 days. Use of FXI/XIa inhibitors was associated with an OR of 0.66 (95% CI = 0.28–1.56) for thromboembolic events (Figure 3). Excluding the study with high risk of bias did not considerably change the results (Supplementary Figure S1). In a sensitivity analysis including data from the EMERALD study, the results remained largely unchanged (Supplementary Figure S2). Because thromboembolic events were not defined as a composite outcome in the EMERALD study, the definitions used in the CONVERT and RE-THINC studies were applied for this analysis (Supplementary Table S7). When comparing the highest dosing of FXI/XIa inhibitor per study to placebo, the ORs for clinically relevant bleeding and thromboembolic events were similar to the main analysis (Supplementary Figure S3).

### Secondary Outcomes

All included studies reported all-cause mortality, whereas only the fully published studies reported rates of major bleeding and clinically relevant nonmajor bleeding. The definition of major bleeding and clinically relevant nonmajor bleeding per study is described in Supplementary Table S7.

**Figure 4:**
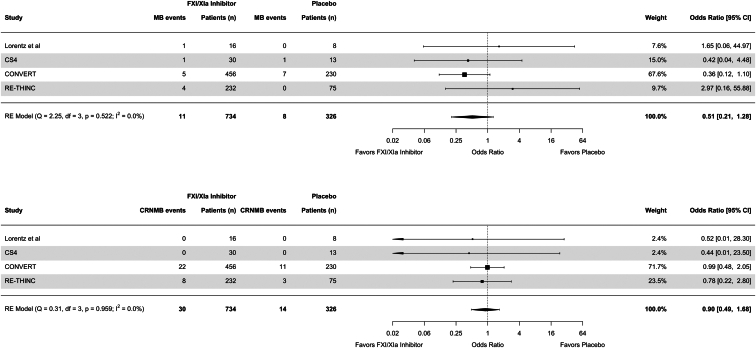
Forest plots for major bleeding and clinically relevant nonmajor bleeding events. CI, confidence interval; CRNMB, clinically relevant nonmajor bleeding; MB, major bleeding; RE, random effects.

**Figure 5:**
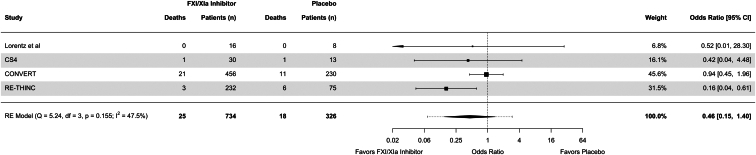
Forest plot for all-cause mortality. CI, confidence interval; RE, random effects.

Use of FXI/XIa inhibitors was associated with an OR of 0.51 (95% CI = 0.21–1.28) for major bleeding and 0.90 (95% CI = 0.49–1.68) for clinically relevant nonmajor bleeding (Figure 4). The respective OR for all-cause mortality was 0.46 (95% CI = 0.15–1.40) (Figure 5). When we excluded the study with high risk of bias, the results were largely unchanged (Supplementary Figures S4 and S5). Similarly, a sensitivity analysis including all-cause mortality rates from the EMERALD study showed comparable point estimates and CIs (Supplementary Figure S6). When comparing the highest dosing groups of FXI/XIa inhibitor per study to placebo, the results were similar to the main analysis, but with considerably increased uncertainty (Supplementary Figures S7 and S8).

## Discussion

In this systematic review and meta-analysis, we provide a summary of available randomized controlled trials investigating FXI/XIa inhibitors in patients with ESKD on HD for the prevention of thromboembolic events. We identified 4 fully published reports and 1 completed study with results posted on ClinicalTrials.gov, all phase 2 trials. Based on compiling the available evidence, the use of FXI/XIa inhibitors was not significantly associated with an increase or decrease in bleeding events, thromboembolic events, or mortality, with a large uncertainty in the estimates.

Notably, all the identified studies on FXI/XIa inhibition included a general HD patient population, without specifically targeting patients with atrial fibrillation, and used placebo as the comparator. This is in contrast to the recently published randomized controlled trials of direct oral anticoagulants, which included specifically patients with ESKD on HD with atrial fibrillation and used vitamin K antagonists as active comparators.^12^, ^13^, ^14^ The rationale for testing FXI/XIa inhibition in patients on HD is rooted in their significantly elevated risk of thromboembolic events, further exacerbated by artificial surfaces of the dialysis system. The pivotal role of contact activation through these artificial surfaces positions patients on HD as a potential target population for this novel class of anticoagulants.^50^^,^^51^ Thus, the prevention of HD system clotting in heparin-free dialysis could also pose a general indication for FXI/XIa inhibitors. Furthermore, the risk of severe bleeding complications in these patients is high, calling for anticoagulants with a favorable safety profile.^8^^,^^16^ The use of currently available anticoagulants in patients with ESKD on HD is still an ongoing debate. Previous research has focused on the subpopulation of patients with atrial fibrillation, with underpowered randomized controlled trials resulting in inconclusive results and a potential net clinical harm of oral anticoagulants observed in cohort studies.^15^^,^^16^^,^^52^^,^^53^ In patients with ESKD on HD without atrial fibrillation, the net clinical effect of anticoagulants for the primary prevention of thromboembolic events is even less clear. Although using placebo as a comparator in the identified studies is therefore a reasonable choice, the application of anticoagulants to all patients with ESKD on HD for the prevention of thromboembolic events is a new clinical approach.

In our synthesis of available data regarding bleeding risk, we did not observe a significant increase or decrease in the odds for bleeding events in patients receiving FXI/XIa inhibitors compared to those with placebo. Regarding clinically relevant bleeding, the 95% CI ranged from a 53% reduction to a 35% increase associated with the use of FXI/XIa inhibitors. This is particularly interesting given that almost all patients received HD system anticoagulation and about half of all patients received low dose aspirin. Further, the results were reassuringly consistent across various bleeding outcomes and sensitivity analyses. Consequently, the currently available evidence on the use of FXI/XIa inhibitors does not indicate a significantly increased bleeding risk in the high-risk group of patients with ESKD on HD, including major bleeding events. Given that the identified studies were not powered to assess efficacy, the results for thromboembolic events must be interpreted with caution. Further, only 2 of the fully published studies and the EMERALD study systematically reported thromboembolic events.^46^, ^47^, ^48^ Both thromboembolic events and all-cause mortality were numerically lower in patients receiving FXI/XIa inhibitors; however, this reduction did not reach statistical significance. Future phase 3 studies are therefore imperative to evaluate the efficacy of FXI/XIa inhibitors in patients with ESKD on HD. Importantly, the number of thromboembolic events was low in both treatment arms compared to previous cohort studies.^16^^,^^51^^,^^54^ This suggests the inclusion of a relatively healthy population of patients, possibly limiting the generalizability to the general cohort of patients with ESKD on HD. Another possible explanation might be the limited follow-up time. Because most patients from the identified studies additionally received HD system anticoagulation, the question of how FXI/XIa inhibitors perform in heparin-free dialysis remains unanswered; however, the safety profile of FXI/XIa inhibitors is promising. We identified 1 ongoing phase 2 trial investigating the FXI/XIa inhibitor MK-2060 compared to placebo, which is planned to be completed in the fourth quarter of 2024. The results of this trial will permit reanalysis of bleeding events, thromboembolic events, and mortality, and might lead to more precise and informative estimates of effect.

In comparing the studies included, several key observations emerged. First, the duration of observation in the study by Lorentz *et al.*^44^ was limited to only 21 days, restricting the ability to capture relevant events. In comparison, the other 4 studies, particularly the CONVERT and RE-THINC studies, had considerably longer observation periods, ranging up to 22 months with median durations of study drug administration of about 9 and 7 months, respectively.^46^^,^^47^ Furthermore, in the study by Lorentz *et al.*,^44^ patients on antiplatelet therapy were excluded and heparin-free dialysis sessions were performed. In the risk of bias assessment, this study was deemed to be at high risk of bias. Therefore, a sensitivity analysis excluding this study was performed, but yielded results consistent with the main analysis. The FXI/XIa inhibitor classes investigated in the identified studies were antibodies and antisense oligonucleotides. This stems from the fact that these drug classes are not cleared via the kidneys and are therefore promising candidates for patients with ESKD on HD.^55^ In addition, the route of administration, that is, injection, of these drug classes might be suitable for patients with ESKD on HD who are attending HD sessions multiple times per week. When considering the mechanism of the investigational drugs included in this systematic review, the mode of action of gruticibart stands out because it binds to factor XI and blocks its activation by factor XIIa, but not by thrombin.^44^^,^^56^^,^^57^ Lastly, it is notable that the EMERALD study has already been completed in 2019, yet we are still awaiting the published report apart from the results which were made available on ClinicalTrials.gov.^48^ Given that the information for the EMERALD study was quite limited, we did not include the results in our main analysis. However, we performed a sensitivity analysis including the results from the EMERALD study, which showed results similar to the main analysis.

In the classical clotting cascade model, the intrinsic pathway, initiated by contact activation, converges with the extrinsic pathway, initiated by exposure of tissue factor to blood, to form the common pathway.^33^^,^^36^ FXI/XIa plays a role in the intrinsic pathway, mediating growth and stabilization of thrombi, and is activated by factor XIIa or thrombin, the latter representing an amplification loop.^33^ However, physiological hemostasis seems to be sufficient without a considerable involvement of the intrinsic pathway. This separation of pathological thrombosis from physiological hemostasis becomes apparent in persons with factor XI deficiency, exhibiting a nonthrombotic phenotype without excessive spontaneous bleeding.^58^ Currently available anticoagulants target thrombin or factor X/Xa, acting more downstream in the coagulation cascade, more specifically in the common pathway.^59^ Thus, these agents are associated with a residual bleeding risk by disrupting physiological hemostasis. Conversely, the inhibition of the intrinsic pathway, e.g., by inhibiting FXI/XIa, represents a promising approach, especially in high-risk patient populations for both bleeding and thromboembolism, such as patients with ESKD on HD.^33^^,^^36^

FXI/XIa inhibitors are currently investigated for a broad variety of clinical applications apart from ESKD on HD, including atrial fibrillation, noncardioembolic stroke, acute coronary syndrome, and treatment of cancer-associated venous thromboembolism.^19^ Five phase 2 trials for these indications have been completed and have shown promising results regarding safety, with some of the studies terminated early due to the considerable reduction in bleeding events.^60^, ^61^, ^62^, ^63^, ^64^ These results informed the design of multiple ongoing phase 3 studies.^19^ One such trial, the OCEANIC-AF study, was discontinued early due to a lack of efficacy, which has raised the first concerns regarding the efficacy of this new class of anticoagulants.^65^ For patients with ESKD on HD, we were able to identify 1 ongoing and 5 completed phase 2 studies. The purpose of phase 2 trials is to investigate safety, that is, bleeding events and adverse events; and for phase 2b trials, to further assess safety and draw first conclusions on efficacy. Although the CONVERT and RE-THINC studies represent phase 2b trials, none of the identified studies were specifically designed and powered to assess efficacy. Further, we were not able to identify any completed or ongoing phase 3 trials. Consequently, it remains unclear whether and when efficacy data will be available for this patient population.

Our systematic review and meta-analysis have some important limitations that are worth mentioning. First, the number of patients included is limited, which is a problem inherent to research in patients with ESKD. However, it was still considerably higher compared to previous randomized controlled trials investigating direct oral anticoagulants in patients with atrial fibrillation and ESKD on HD.^12^, ^13^, ^14^ Furthermore, the numbers of events were low, especially in the smaller scale studies. Whereas bleeding was reported consistently in all included studies, thromboembolic events were only reported in CONVERT, RE-THINC, and EMERALD. Importantly, the design of the included studies was heterogeneous, with differences in setting, primary outcome, number of included patients, and duration of observation. We could not perform subgroup analyses by type of FXI/XIa inhibitor and were not able to evaluate publication bias. However, for all registered and completed studies, results were available. Lastly, we only identified phase 2 trials, limiting the availability to draw conclusions about efficacy outcomes.

In summary, currently available evidence on the use of FXI/XIa inhibitors in patients with ESKD on HD does not indicate a significantly increased risk of bleeding compared to placebo. The efficacy of these inhibitors regarding the reduction of thromboembolic events and their impact on mortality remain to be evaluated in sufficiently powered, randomized controlled phase 3 trials.

## Disclosure

DK has received honoraria for participation in advisory boards from CSL Behring. OK has received honoraria for participation in advisory boards from BMS and Pfizer. CA has received honoraria for lectures and/or participation in advisory boards from Bayer, BMS, Pfizer, Daiichi Sankyo, Sanofi. All the other authors declared no competing interests.

## Supplementary Material

Supplementary File (PDF) **Table S1.** PRISMA 2020 checklist. **Table S2.** Search strategies. **Table S3.** Ongoing phase 2 and completed phase 1 studies. **Table S4.** Detailed in- and exclusion criteria of included studies. **Table S5.** Baseline characteristics of randomized patients. **Table S6.** Risk of bias assessment using the revised Cochrane risk of bias tool (ROB 2). **Table S7.** Definitions of bleeding and thromboembolic events. **Figure S1.** Forest plot for clinically relevant bleeding (CRB) events excluding the study by Lorentz *et al.***Figure S2.** Forest plots for clinically relevant bleeding (CRB) and thromboembolic (TE) events including EMERALD. **Figure S3.** Forest plots for clinically relevant bleeding (CRB) and thromboembolic (TE) events comparing the highest dosing groups of FXI/XIa inhibitors per study to placebo. **Figure S4.** Forest plots for major bleeding (MB) and clinically relevant non-major bleeding (CRNMB) events excluding the study by Lorentz *et al.***Figure S5.** Forest plot for all-cause mortality excluding the study by Lorentz *et al.***Figure S6.** Forest plot for all-cause mortality including EMERALD. **Figure S7.** Forest plots for major bleeding (MB) and clinically relevant non-major bleeding (CRNMB) events comparing the highest dosing groups of FXI/XIa inhibitors per study to placebo. **Figure S8.** Forest plot for all-cause mortality comparing the highest dosing groups of FXI/XIa inhibitors per study to placebo.
