# Supplementary material for: A Systematic Review of Safety and Efficacy of Factor XI/XIa Inhibitors in Patients With ESKD on Hemodialysis
Source: Kidney Int Rep. 2024 Oct 15;10(1):145–56. doi: 10.1016/j.ekir.2024.10.007 (PMC11725973; doi:10.1016/j.ekir.2024.10.007)
Supplement: Supplementary File (PDF) — Table S1. PRISMA 2020 checklist. Table S2. Search strategies. Table S3. Ongoing phase 2 and completed phase 1 studies. Table S4. Detailed in- and exclusion criteria of included studies. Table S5. Baseline characteristics of randomized patients. Table S6. Risk of bias assessment using the revised Cochrane risk of bias tool (ROB 2). Table S7. Definitions of bleeding and thromboembolic events. Figure S1. Forest plot for clinically relevant bleeding (CRB) events excluding the study by Lorentz et al.Figure S2. Forest plots for clinically relevant bleeding (CRB) and thromboembolic (TE) events including EMERALD. Figure S3. Forest plots for clinically relevant bleeding (CRB) and thromboembolic (TE) events comparing the highest dosing groups of FXI/XIa inhibitors per study to placebo. Figure S4. Forest plots for major bleeding (MB) and clinically relevant non-major bleeding (CRNMB) events excluding the study by Lorentz et al.Figure S5. Forest plot for all-cause mortality excluding the study by Lorentz et al.Figure S6. Forest plot for all-cause mortality including EMERALD. Figure S7. Forest plots for major bleeding (MB) and clinically relevant non-major bleeding (CRNMB) events comparing the highest dosing groups of FXI/XIa inhibitors per study to placebo. Figure S8. Forest plot for all-cause mortality comparing the highest dosing groups of FXI/XIa inhibitors per study to placebo. [file mmc1.pdf]

Table of Contents

Table S1: PRISMA 2020 checklist 2

Table S2: Search strategies 4

Table S3: Ongoing phase 2 and completed phase 1 studies 7

Table S4: Detailed in- and exclusion criteria of included studies 8

Table S5: Baseline characteristics of randomized patients 15

Table S6: Risk of bias assessment using the revised Cochrane risk of bias tool (ROB 2) 16

Table S7: Definitions of bleeding and thromboembolic events 17

Figure S1: Forest plot for clinically relevant bleeding (CRB) events excluding the study by Lorentz et al 19

Figure S2: Forest plots for clinically relevant bleeding (CRB) and thromboembolic (TE) events including EMERALD 20

Figure S3: Forest plots for clinically relevant bleeding (CRB) and thromboembolic (TE) events comparing the highest dosing groups of FXI/XIa inhibitors per study to placebo 21

Figure S4: Forest plots for major bleeding (MB) and clinically relevant non-major bleeding (CRNMB) events excluding the study by Lorentz et al 22

Figure S5: Forest plot for all-cause mortality excluding the study by Lorentz et al 23

Figure S6: Forest plot for all-cause mortality including EMERALD 24

Figure S7: Forest plots for major bleeding (MB) and clinically relevant non-major bleeding (CRNMB) events comparing the highest dosing groups of FXI/XIa inhibitors per study to placebo 25

Figure S8: Forest plot for all-cause mortality comparing the highest dosing groups of FXI/XIa inhibitors per study to placebo 26

Table S1: PRISMA 2020 checklist

			**Location where item is reported**
**TITLE**			
Title	1	Identify the report as a systematic review.	Title
**ABSTRACT**			
Abstract	2	See the PRISMA 2020 for Abstracts checklist.	Abstract
**INTRODUCTION**			
Rationale	3	Describe the rationale for the review in the context of existing knowledge.	Introduction
Objectives	4	Provide an explicit statement of the objective(s) or question(s) the review addresses.	Introduction
**METHODS**			
Eligibility criteria	5	Specify the inclusion and exclusion criteria for the review and how studies were grouped for the syntheses.	Methods
Information sources	6	Specify all databases, registers, websites, organisations, reference lists and other sources searched or consulted to identify studies. Specify the date when each source was last searched or consulted.	Methods
Search strategy	7	Present the full search strategies for all databases, registers and websites, including any filters and limits used.	Supplement
Selection process	8	Specify the methods used to decide whether a study met the inclusion criteria of the review, including how many reviewers screened each record and each report retrieved, whether they worked independently, and if applicable, details of automation tools used in the process.	Methods
Data collection process	9	Specify the methods used to collect data from reports, including how many reviewers collected data from each report, whether they worked independently, any processes for obtaining or confirming data from study investigators, and if applicable, details of automation tools used in the process.	Methods
Data items	10a	List and define all outcomes for which data were sought. Specify whether all results that were compatible with each outcome domain in each study were sought (e.g. for all measures, time points, analyses), and if not, the methods used to decide which results to collect.	Methods
	10b	List and define all other variables for which data were sought (e.g. participant and intervention characteristics, funding sources). Describe any assumptions made about any missing or unclear information.	Protocol
Study risk of bias assessment	11	Specify the methods used to assess risk of bias in the included studies, including details of the tool(s) used, how many reviewers assessed each study and whether they worked independently, and if applicable, details of automation tools used in the process.	Methods
Effect measures	12	Specify for each outcome the effect measure(s) (e.g. risk ratio, mean difference) used in the synthesis or presentation of results.	Methods
Synthesis methods	13a	Describe the processes used to decide which studies were eligible for each synthesis (e.g. tabulating the study intervention characteristics and comparing against the planned groups for each synthesis (item #5)).	Methods
	13b	Describe any methods required to prepare the data for presentation or synthesis, such as handling of missing summary statistics, or data conversions.	Methods
	13c	Describe any methods used to tabulate or visually display results of individual studies and syntheses.	Methods
	13d	Describe any methods used to synthesize results and provide a rationale for the choice(s). If meta-analysis was performed, describe the model(s), method(s) to identify the presence and extent of statistical heterogeneity, and software package(s) used.	Methods
	13e	Describe any methods used to explore possible causes of heterogeneity among study results (e.g. subgroup analysis, meta-regression).	Methods
	13f	Describe any sensitivity analyses conducted to assess robustness of the synthesized results.	Methods
Reporting bias assessment	14	Describe any methods used to assess risk of bias due to missing results in a synthesis (arising from reporting biases).	Methods
Certainty assessment	15	Describe any methods used to assess certainty (or confidence) in the body of evidence for an outcome.	Methods
**RESULTS**			
Study selection	16a	Describe the results of the search and selection process, from the number of records identified in the search to the number of studies included in the review, ideally using a flow diagram.	Results, figure 1

	16b	Cite studies that might appear to meet the inclusion criteria, but which were excluded, and explain why they were excluded.	na
Study characteristics	17	Cite each included study and present its characteristics.	Results, table 1
Risk of bias in studies	18	Present assessments of risk of bias for each included study.	Results, supplement
Results of individual studies	19	For all outcomes, present, for each study: (a) summary statistics for each group (where appropriate) and (b) an effect estimate and its precision (e.g. confidence/credible interval), ideally using structured tables or plots.	Figures 2-5, supplement
Results of syntheses	20a	For each synthesis, briefly summarise the characteristics and risk of bias among contributing studies.	Results
	20b	Present results of all statistical syntheses conducted. If meta-analysis was done, present for each the summary estimate and its precision (e.g. confidence/credible interval) and measures of statistical heterogeneity. If comparing groups, describe the direction of the effect.	Results, figures 2-5
	20c	Present results of all investigations of possible causes of heterogeneity among study results.	Figures 2-5
	20d	Present results of all sensitivity analyses conducted to assess the robustness of the synthesized results.	Supplement
Reporting biases	21	Present assessments of risk of bias due to missing results (arising from reporting biases) for each synthesis assessed.	na
Certainty of evidence	22	Present assessments of certainty (or confidence) in the body of evidence for each outcome assessed.	Results, figures 2-5
**DISCUSSION**			
Discussion	23a	Provide a general interpretation of the results in the context of other evidence.	Discussion
	23b	Discuss any limitations of the evidence included in the review.	Discussion
	23c	Discuss any limitations of the review processes used.	Discussion
	23d	Discuss implications of the results for practice, policy, and future research.	Discussion
**OTHER INFORMATION**			
Registration and protocol	24a	Provide registration information for the review, including register name and registration number, or state that the review was not registered.	Methods
	24b	Indicate where the review protocol can be accessed, or state that a protocol was not prepared.	Methods
	24c	Describe and explain any amendments to information provided at registration or in the protocol.	na
Support	25	Describe sources of financial or non-financial support for the review, and the role of the funders or sponsors in the review.	No funding
Competing interests	26	Declare any competing interests of review authors.	Disclosure
Availability of data, code and other materials	27	Report which of the following are publicly available and where they can be found: template data collection forms; data extracted from included studies; data used for all analyses; analytic code; any other materials used in the review.	Data availability statement

Table S2: Search strategies

MEDLINE®

**#19**	**#4 AND #7 AND #18**
#18	#16 NOT #17
#17	animals [mh] NOT humans [mh]
#16	#8 OR #9 OR #10 OR #11 OR #12 OR #13 OR #14 OR #15
#15	groups [tiab]
#14	trial [tiab]
#13	randomly [tiab]
#12	drug therapy [sh]
#11	placebo [tiab]
#10	randomized [tiab]
#9	controlled clinical trial [pt]
#8	randomized controlled trial [pt]
#7	#5 OR #6
#6	“factor XI*”[tiab] OR “f xi*”[tiab] OR fxi*[tiab] OR “factor 11*”[tiab] OR “f 11*”[tiab] OR f11*[tiab] OR milvexian*[tiab] OR BMS-986177*[tiab] OR JNJ-70033093*[tiab] OR asundexian*[tiab] OR “BAY 2433334*”[tiab] OR osocimab*[tiab] OR “BAY 1213790*”[tiab] OR abelacimab*[tiab] OR MAA868*[tiab] OR xisomab*[tiab] OR AB023*[tiab] OR IONIS-FXIRx*[tiab] OR BAY2306001*[tiab] OR ISIS-416858*[tiab] OR ISIS-404071*[tiab] OR FXI-LICA*[tiab] OR BAY 2976217*[tiab] OR fesomersen*[tiab] OR MK-2060*[tiab] OR gruticibart*[tiab] OR BAY-1831865*[tiab] OR ONO-7648*[tiab] OR SHR-2285*[tiab] OR BMS-962212*[tiab] OR EP-7041*[tiab] OR Ir-CPI*[tiab] OR fasxiator*[tiab] OR acaNAP10*[tiab] OR desmolaris*[tiab] OR boophilin*[tiab] OR FELIAP*[tiab]
#5	„factor XI”[MeSH Terms]
#4	#1 OR #2 OR #3
#3	hemodialyis*[tiab] OR haemodialysis*[tiab] OR hemodiafiltration*[tiab] OR haemodiafiltration*[tiab] OR kidney*[tiab] OR renal*[tiab]
#2	„kidney failure, chronic“[MeSH Terms]
#1	„renal replacement therapy“[MeSH Terms]

Embase

**#46**	**#5 AND #10 AND #45**
#45	#30 NOT #44
#44	#31 OR #32 OR #33 OR #34 OR #35 OR #36 OR #37 OR #38 OR #39 OR #40 OR #41 OR #42 OR #43
#43	(‘animal experiment’/de NOT (‘human experiment’/de OR ‘human’/de))
#42	((rat:ti,tt OR rats:ti,tt OR mouse:ti,tt OR mice:ti,tt OR swine:ti,tt OR porcine:ti,tt OR murine:ti,tt OR sheep:ti,tt OR lambs:ti,tt OR pigs:ti,tt OR piglets:ti,tt OR rabbit:ti,tt OR rabbits:ti,tt OR cat:ti,tt OR cats:ti,tt OR dog:ti,tt OR dogs:ti,tt OR cattle:ti,tt OR bovine:ti,tt OR monkey:ti,tt OR monkeys:ti,tt OR trout:ti,tt OR marmoset*:ti,tt) AND ‘animal experiment’/de)
#41	(databases NEAR/5 searched):ab
#40	‘update review’:ab

#39	(‘we searched’:ab AND (review:ti,tt OR review:it))
#38	(review:ab AND review:it) NOT trial:ti,tt
#37	(‘random cluster’ NEAR/4 sampl*):ti,ab,tt
#36	‘random field*’:ti,ab,tt
#35	(nonrandom*:ti,ab,tt NOT random*:ti,ab,tt)
#34	(‘systematic review’:ti,tt NOT (trial:ti,tt OR study:ti,tt))
#33	(‘case control*’:ti,ab,tt AND random*:ti,ab,tt NOT (‘randomised controlled’:ti,ab,tt OR ‘randomized controlled’:ti,ab,tt))
#32	(‘cross-sectional study’/de NOT (‘randomized controlled trial’/exp OR ‘controlled clinical study’/de OR ‘controlled study’/de OR ‘randomised controlled’:ti,ab,tt OR ‘randomized controlled’:ti,ab,tt OR ‘control group’:ti,ab,tt OR ‘control groups’:ti,ab,tt))
#31	(((random* NEXT/1 sampl* NEAR/8 (‘cross section*’ OR questionnaire* OR survey OR surveys OR database or databases)):ti,ab,tt) NOT (‘comparative study’/de OR ‘controlled study’/de OR ‘randomised controlled’:ti,ab,tt OR ‘randomized controlled’:ti,ab,tt OR ‘randomly assigned’:ti,ab,tt))
#30	#11 OR #12 OR #13 OR #14 OR #15 OR #16 OR #17 OR #18 OR #19 OR #20 OR #21 OR #22 OR #23 OR #24 OR #25 OR #26 OR #27 OR #28 OR #29
#29	trial:ti,tt
#28	‘human experiment’/de
#27	(volunteer:ti,ab,tt OR volunteers:ti,ab,tt)
#26	(controlled NEAR/8 (study OR design OR trial)):ti,ab,tt
#25	(assigned:ti,ab,tt OR allocated:ti,ab,tt)
#24	((assign* OR match OR matched OR allocation) NEAR/6 (alternate OR group OR groups OR intervention OR interventions OR patient OR patients OR subject OR subjects OR participant OR participants)):ti,ab,tt
#23	(crossover:ti,ab,tt OR ‘cross over’:ti,ab,tt)
#22	(parallel NEXT/1 group*):ti,ab,tt
#21	‘double blind procedure’/de
#20	((double OR single OR doubly OR singly) NEXT/1 (blind OR blinded OR blindly)):ti,ab,tt
#19	(open NEXT/1 label):ti,ab,tt
#18	((evaluated:ab OR evaluate:ab OR evaluating:ab OR assessed:ab OR assess:ab) AND (compare:ab OR compared:ab OR comparing:ab OR comparison:ab))
#17	(compare:ti,tt OR compared:ti,tt OR comparison:ti,tt)
#16	placebo:ti,ab,tt
#15	‘intermethod comparison’/de
#14	‘randomization’/de
#13	random*:ti,ab,tt
#12	‘controlled clinical trial’/de
#11	‘randomized controlled trial’/exp
#10	#6 OR #7 OR #8 OR #9
#9	(‘factor xi*’ OR ‘f xi*’ OR fxi* OR ‘factor 11*’ OR ‘f 11*’ OR f11* OR milvexian* OR BMS-986177* OR JNJ-70033093* OR asundexian* OR ‘BAY 2433334*’ OR osocimab* OR ‘BAY 1213790*’ OR abelacimab* OR MAA868* OR xisomab* OR AB023* OR IONIS-FXIRx* OR BAY2306001* OR ISIS-416858* OR ISIS-404071* OR FXI-LICA* OR ‘BAY 2976217*‘ OR fesomersen* OR MK-2060* OR gruticibart* OR BAY-1831865* OR ONO-7648* OR SHR-2285* OR BMS-962212* OR EP-7041* OR Ir-CPI* OR fasxiator* OR acaNAP10* OR desmolaris* OR boophilin* OR FELIAP*):ti,ab,kw
#8	‘blood clotting factor 11’/exp

#7	‘blood clotting factor 11a’/exp
#6	‘blood clotting factor 11a inhibitor’/exp
#5	#1 OR #2 OR #3 OR #4
#4	(h$emodialysis* OR h$emodiafiltration* OR renal* OR kidney*):ti,ab,kw
#3	‚renal replacement therapy‘/exp
#2	‚dialysis‘/exp
#1	‘end-stage renal disease’/exp

ClinicalTrials.gov

**1**	**Condition**
	Hemodialysis OR Haemodialysis OR Hemodiafiltration OR Haemodiafiltration OR Renal OR Kidney
**2**	**AND Intervention**
	Factor XI OR F XI OR FXI OR Factor 11 OR F 11 OR F11 OR Factor XIa OR F XIa OR FXIa OR Factor 11a OR F 11a OR F11a OR Milvexian OR BMS-986177 OR JNJ-70033093 OR Asundexian OR BAY 2433334 OR Osocimab OR BAY 1213790 OR Abelacimab OR MAA868 OR Xisomab OR AB023 OR IONIS-FXIRx OR BAY2306001 OR ISIS-416858 OR ISIS-404071 OR FXI-LICA OR BAY 2976217 OR Fesomersen OR MK-2060 OR Gruticibart OR BAY-1831865 OR ONO-7648 OR SHR-2285 OR BMS-962212 OR EP-7041 OR Ir-CPI OR Fasxiator OR acaNAP10 OR Desmolaris OR Boophilin OR FELIAP

Table S3: Ongoing phase 2 and completed phase 1 studies

	Official title	Design	Patients	Intervention	Control	Primary endpoints	Completion date
**Ongoing (phase 2)**	-	-	-	-	-	-	-
NCT05027074	A Randomized Parallel-group, Placebo-controlled, Double-blind, Event-driven, Multi-center Phase II Clinical Outcome Trial of Prevention of Arteriovenous Graft Thrombosis and Safety of MK-2060 in Patients With End Stage Renal Disease Receiving Hemodialysis	Randomized, blinded	On hemodialysis or hemodiafiltration ≥3 times per week for a minimum of 3 hour/session, uninfected AVG (n=489)	MK-2060	Placebo	AV graft thrombosis	2024-10-31 (planned)
**Completed (phase 1)**	-	-	-	-	-	-	-
NCT03787368	An Observer-blind, Multi-center, Placebo-controlled, Parallel Group Study to Assess the Safety and Tolerability and to Characterize the Pharmacokinetics and the Pharmacodynamics of Different Doses of BAY1213790 in Patients With End-stage Renal Disease Undergoing Hemodialysis	Randomized, blinded	On hemodialysis >3 months, between 18 and 80 years of age (n=55)	Osocimab	Placebo	Major and CRNMB	2021-09-15
NCT03873038	Single and Multiple Dose Clinical Trial to Study the Safety and Pharmacokinetics of MK-2060 in Older Participants With End-Stage Renal Disease on Hemodialysis	Randomized, sequential assignment, blinded	On hemodialysis for >3 months 3 times/week with 3 hours/session, age ≥40 and ≤80 years for part 1 and ≥18 and ≤80 years for part 2 (n=38)	MK-2060	Placebo	Adverse events	2021-12-20
NCT05769595	A Single-dose Clinical Study to Evaluate the Safety, Tolerability, Pharmacokinetics and Pharmacodynamics of MK-2060 in Japanese Older Participants With End-stage Renal Disease on Dialysis.	Randomized, double-blind	Japanese descent, on hemodialysis or hemodiafiltration, 50 to 80 years (n=17)	MK-2060	Placebo	Adverse events	2024-02-15

Table S4: Detailed in- and exclusion criteria of included studies

	Lorentz et al	CS4	CONVERT	RE-THINC	EMERALD
**Inclusion**	18 to 80 years of age, end-stage renal disease on a stable, 3-times-per-week outpatient hemodialysis regimen for >3 months utilizing arteriovenous (AV) fistula or AV graft BMI of ≥ 18 at the time of screening.<br>Hemodialysis stability defined as Kt/V ≥ 1.2 within 3 months prior to screening at a healthcare center for > 3 months from screening.<br>Capability of understanding the written informed consent, providing signed and witnessed written informed consent, and agreeing to comply with protocol requirements and study related procedures.<br>Willing to be confined to the clinical research unit for the duration of the study, able to comply with all study-related requirements, and able to adhere to study restrictions and visit schedules.<br>Considered by the PI to be clinically stable with	18 to 80 years of age with end-stage renal disease who had been receiving chronic HD with heparin 3 times per week for a minimum of 3 hours per dialysis session for at least 3 months	≥18 years of age, end-stage kidney disease undergoing hemodialysis (including hemodiafltration) for at least 9 hours per week ≥3 months and stable, in the view of the investigator, body weight ≥50 kg, contraceptive use consistent with local regulations regarding the methods of contraception for those participating in clinical studies, capable of providing signed informed consent	18 years or older with kidney failure on thrice-weekly hemodialysis for at least 9 hours per week and for at least 3 months and willing to adhere to the study procedures, contraceptive use by men or women should be consistent with local regulations regarding the methods of contraception for those participating in clinical studies, capable of giving signed ICF as described in the Protocol, which includes compliance with the requirements and restrictions listed in the ICF and in the protocol.	18 to 85 years of age with end-stage renal disease maintained on outpatient hemodialysis at a healthcare center for > 3 months from screening with hemodialysis at least 3 times per week for a minimum of 9 hours per week of prescribed treatment time and plan to continue this throughout the study.

	respect to underlying ESRD, based on medical evaluation that includes medical and surgical history, and a complete physical examination including vital signs, ECG, and clinical laboratory test results at screening.<br>Female patients must be of non-childbearing potential and must have undergone one of the following: sterilization procedures at least 6 months prior to dosing: hysteroscopic sterilization; bilateral tubal ligation or bilateral salpingectomy; hysterectomy; bilateral oophorectomy; or be postmenopausal with amenorrhea for at least 1 year prior to dosing and follicle stimulating hormone (FSH) serum levels consistent with postmenopausal status as per PI or designee judgment.<br>Male patients must either be sterile (vasectomy with history of a negative sperm count following the procedure); practice total abstinence from sexual intercourse as the				

	preferred lifestyle (periodic abstinence is not acceptable); use a male condom with any sexual activity; or agree to use a birth control method considered to be appropriate by the Investigator (such as one of the methods identified above for female patients) from the time of screening until 90 days after study drug administration. Male patients must agree not to donate sperm for a period of 90 days after study drug administration.				
**Exclusion**	Dialysis access via a catheter; continuous anticoagulation and/or antiplatelet therapy (apart from HD system anticoagulation); history of venous or arterial thromboembolic events (acute coronary syndrome, stroke or transient ischemic attack, venous thromboembolic event) within the preceding 3 months; platelet count <75 x 10^9/L, international normalized ratio >1.4, aPTT >1.5 times the upper limit of normal.	Recent thrombotic (acute coronary syndrome, stroke or transient ischemic attack, venous thromboembolic event) or bleeding event in the past 3 months, an abnormal coagulation profile or elevated liver enzymes (platelet count < 150,000 cells/mm^3, INR > 1.4 aPTT > upper limit of normal (ULN), ALT or AST > 2 x ULN, total bilirubin > ULN), concomitant use of an anticoagulant or antiplatelet agent other than HD circuit anticoagulation (e.g.,	Recent (<6 months before screening) clinically significant bleeding, hemoglobin <9.0 g/dl, platelet count <100 × 10^9/l, activated partial thromboplastin time or prothrombin time above the upper limit of normal, hepatic disease associated with alanine aminotransferase over three times the upper limit of normal, or total bilirubin over two times the upper limit of normal with direct bilirubin over 20% of the total, sustained uncontrolled	Recent (<6 months before screening) clinically significant bleeding, at high risk of bleeding (in the judgement of the investigator), known bleeding disorders, recent (<3 months before screening) major surgery or scheduled major surgery, platelet count < 100 x 10^9/l, liver disease (alanine transaminase > 3 times the upper limit of normal, or total bilirubin > 2 times the upper limit of normal with direct bilirubin >20% of the total), recent (<3 months	History of major medical event (previous acute coronary syndrome, stroke or transient ischemic attack or systemic thromboembolic event) within 3 months of screening, major surgery within 3 months of screening, or new major physical examination finding except for documented atrial fibrillation, active bleeding within the past 3 months from screening or documented bleeding diathesis (excluding uremia), coagulopathy, or

	Any clinically significant (CS) concomitant disease or condition (including treatment for such conditions) that, in the opinion of the PI, could either interfere with the study drug, compromise interpretation of study data, or pose an unacceptable risk to the patient.<br>Any other CS abnormalities in laboratory test results at screening that would, in the opinion of the PI, increase the patient's risk of participation, jeopardize complete participation in the study, or compromise interpretation of study data.<br>Pregnant (positive pregnancy test) at screening or check-in on Day -8. If serum human chorionic gonadotropin (hCG) pregnancy test results are indeterminate, follow-up testing should be performed to determine eligibility.<br>Treatment with another investigational drug or device study within 30 days (or 5 half lives,	heparin) or low-dose aspirin (<100 mg), life expectancy <1 year, uncontrolled hypertension as judged by the Investigator, a pre- or post-dialysis blood pressure (BP) > 160 mmHg on at least 3 of last 5 dialysis treatments, planned major surgery in the next 6 months (e.g. renal transplant surgery)	hypertension (diastolic blood pressure ≥100 mmHg and/or systolic blood pressure ≥180 mmHg), known intracranial neoplasm, arteriovenous malformation or aneurysm, known bleeding disorders, recent (<3 months before screening) thromboembolic event (e.g. acute coronary syndrome, stroke or VTE (except dialysis access thrombosis)), recent (<3 months before screening) major surgery or scheduled major surgery during study participation, scheduled living donor renal transplant during study participation, persistent heart failure, as classifed by the New York Heart Association classifcation of III or higher, receiving antiplatelet therapy, except acetylsalicylic acid ≤150 mg per day, receiving anticoagulation in therapeutic doses, other than standard anticoagulation during the hemodialysis procedure, life expectancy	before screening) thromboembolic event (e.g. acute coronary syndrome, stroke, or venous thromboembolism (except dialysis access thrombosis)), persistent heart failure (New York Heart Association class III or higher), sustained uncontrolled hypertension (persistent measurements of diastolic blood pressure ≥ 100 mmHg, and/or systolic blood pressure ≥ 180 mmHg), active malignancy, life expectancy of less than 6 months, receiving antiplatelet therapy (except acetylsalicylic acid ≤150 mg) or anticoagulation in therapeutic doses (other than standard anticoagulation during the hemodialysis procedure, scheduled living donor renal transplant during study participation, known Hepatitis B or C, known HIV with recent documented detectable viral load (<3 months before screening), hemoglobin < 9.0 g/dL at screening, known	recent prolonged compression time at arteriovenous fistula, platelet count < 150,000 cells/mm^3, < 180,000 cells/mm^3 for platelet function/activation subgroup, international normalized ratio (INR) > 1.4, activated partial thromboplastin time (aPTT) > upper limit of normal (ULN), alanine aminotransferase (ALT) or aspartate aminotransferase (AST) > 2 x ULN, total bilirubin > ULN, factor XI (FXI) activity < 0.3 units per milliliter (U/mL), malignancy within 5 years, except basal or squamous cell carcinoma of the skin or carcinoma in situ of the cervix that has been successfully treated. Participants with malignancies that have been treated with curative intent and which have no reoccurrence within 5 years may also be eligible if approved by Sponsor Medical Monitor.<br>Any of the following within 6 months prior to screening: More than 3 episodes of severe hypoglycemia requiring

	whichever is longer) prior to check-in on Day -8.<br>Acute illness that is considered by the PI to be CS within 2 weeks of check-in on Day 8.<br>Currently have established underlying inherited or acquired symptomatic bleeding disorders and/or are at risk for excessive bleeding per PI judgment or current active bleeding (e.g., gastrointestinal, intracranial), aside from minor bleeding from the puncture site on the AV fistula or AV graft, which would be expected to occur during the dialysis procedure, with the following values:<br>ALT or AST > 2 x ULN at screening<br>Total bilirubin > 1.2 ULN at screening<br>Hemoglobin concentration < 10 g/dL at screening<br>Seated blood pressure < 90/40 mmHg at screening and check-in on Day -8.<br>Exclusion criteria for ECG at screening and check-in on Day -8:<br>Heart rate < 45 and > 110 bpm QTcF interval > 500		<6 months, active malignancy requiring treatment during study participation (except nonmelanoma skin cancer or cervical carcinoma in situ), known hypersensitivity to the investigational drug or to inactive constituents of the study drug, participation in another clinical study with an investigational medicinal product within 30 days or within five half-lives of such, whichever is longer, before randomization and during the study, any other conditions, which, in the opinion of the investigator<br>or sponsor, would render the individual unsuitable for inclusion	hypersensitivity to the investigational drug or to inactive constituents of the study intervention, participation in a study with an investigational medicinal product within 30 days or within 5 half-lives of the previous administered drug, whichever is longer, prior to the screening/observational period (Note: Participants from previous BAY2306001/ISIS 416858 and BAY2976217/ ION 957943 studies were eligible), confirmed pregnancy, any other conditions, which, in the opinion of the investigator or Sponsor would make the subject unsuitable for inclusion.	the assistance of another person to actively administer carbohydrate, glucagon or other resuscitative actions, one event of hypoglycemia in which the participant required hospitalization, recurrent syncope and recurrent hypotension in the inter-dialytic period requiring intervention.<br>Planned major surgery in the next 6 months, including participants receiving kidney transplant or participants that anticipate changing dialysis modality (i.e. hemodialysis to peritoneal dialysis), concomitant use of anticoagulant or antiplatelet agents (e.g., warfarin, dabigatran, rivaroxaban, clopidogrel) that may affect coagulation (except low dose aspirin (≤ 100 mg/day) during Treatment and Post-treatment Evaluation Periods. Stable does of heparins during dialysis permitted.<br>Uncontrolled hypertension as judged by the Investigator.<br>Participants with a pre- or post-dialysis blood

	msec (bpm = beats per minute; msec = milliseconds; QTcF = QT interval corrected using Fridericia's formula)<br><br>Any significant arrhythmia or conduction abnormality, (including but not specific to atrioventricular block [2nd degree or higher], Wolff Parkinson White syndrome [unless curative radio ablation therapy]), which, in the opinion of the PI and Medical Monitor, could interfere with the safety for the individual patient.<br>Non-sustained or sustained ventricular tachycardia (> 2 consecutive ventricular ectopic beats at a rate of > 1.7/second).<br>History of a CS allergy to recombinant biologic drug, rodents, or a known sensitivity or idiosyncratic reaction to any compound present in xisomab 3G3, its related compounds, or any compound listed as being present in the study formulation.<br>Participate in strenuous exercise from 48 hours				pressure (BP) that is > 180 millimeters of mercury (mmHg) on at least 3 of last 5 dialysis treatments.

	prior to check-in on Day -8 and throughout the study.<br>Positive test for drugs of abuse and/or positive alcohol test at screening or check in on Day 8 if not accounted for by a prescription medication. Patients with a positive test based on a prescribed medication may be enrolled.<br>Positive test at screening for hepatitis B surface antigen (HBsAg) or human immunodeficiency virus (HIV). If a patient with ESRD has positive test results for hepatitis C virus (HCV) but liver function tests are otherwise not clinically significant, the patient may be included at the PI's discretion.<br>Receiving blood purification therapy other than HD.<br>Any other reason that would render the patient unsuitable for study enrollment at the discretion of the PI.				

Table S5: Baseline characteristics of randomized patients

Study (intervention)	Lorentz et al (Gruticibart)			CS4 (IONIS-FXI$_{Rx}$)			CONVERT (Osocimab)			RE-THINC (Fesomersen)				EMERALD (IONIS-FXI$_{Rx}$)			
**Dose**	**0.25 mg/kg (n=8)**	**0.50 mg/kg (n=8)**	**Placebo (n=8)**	**200 mg (n=15)**	**300 mg (n=15)**	**Placebo (n=13)**	**105 mg, 52.5 mg (n=232)**	**210 mg, 105 mg (n=224)**	**Placebo (n=230)**	**40 mg (n=77)**	**80 mg (n=79)**	**120 mg (n=76)**	**Placebo (n=75)**	**200 mg (n=53)**	**250 mg (n=54)**	**300 mg (n=50)**	**Placebo (n=53)**
**Age, yr, mean (SD)**	55.8 (7.6)	53.4 (5.0)	52.5 (9.2)	-	-	-	-	-	-	60.4 (13.2)	58.0 (14.4)	57.7 (13.3)	58.6 (11.9)	61 (14)	63 (12)	58 (14)	61 (13)
**Age, yr, median (range)**	-	-	-	56 (31-77)	58 (29-76)	63 (40-80)	61 (28-91)	61 (25-90)	60 (24-90)	-	-	-	-	-	-	-	-
**Women, n (%)**	1 (12.5)	3 (37.5)	1 (12.5)	5 (33.3)	12 (80.0)	4 (30.8)	89 (38.4)	81 (36.2)	80 (34.8)	35 (45.5)	20 (25.3)	30 (39.5)	22 (29.3)	23 (43.4)	19 (35.2)	20 (40.0)	19 (35.8)
**BMI, kg/m², mean (SD)**	27.8 (3.9)	27.9 (5.4)	24.7 (2.9)	nr	nr	nr	-	-	-	-	-	-	-	nr	nr	nr	nr
**BMI >30 kg/m², n (%)**	-	-	-	nr	nr	nr	74 (31.9)	65 (29.0)	62 (27.0)	19 (24.7)	20 (25.3)	27 (35.5)	18 (24.0)	nr	nr	nr	nr
**Etiology of kidney disease, n (%)**	-	-	-	-	-	-	-	-	-	-	-	-	-	-	-	-	-
Hypertension	6 (75)	5 (62.5)	4 (50)	0 (0)	1 (6.7)	2 (15.4)	51 (22.0)	67 (30.0)	50 (21.7)	14 (18.2)	14 (17.7)	19 (25.0)	15 (20.0)	nr	nr	nr	nr
Diabetes	0	2 (25)	0 (0)	5 (33.3)	8 (53.3)	5 (38.5)	65 (28.0)	53 (23.6)	59 (25.6)	14 (18.2)	21 (26.6)	19 (25.0)	19 (25.3)	nr	nr	nr	nr
Glomerulonephritis	nr	nr	nr	4 (26.7)	2 (13.3)	2 (15.4)	32 (13.8)	30 (13.4)	33 (14.3)	13 (16.9)	14 (17.7)	16 (21.1)	9 (12.0)	nr	nr	nr	nr
Polycystic kidney disease	1 (12.5)	0 (0)	0 (0)	2 (13.3)	0 (0)	0 (0)	23 (9.9)	23 (10.3)	21 (9.1)	10 (13.0)	11 (13.9)	1 (1.3)	8 (10.7)	nr	nr	nr	nr
Pyelonephritis	nr	nr	nr	nr	nr	nr	11 (4.7)	10 (4.5)	10 (4.3)	nr	nr	nr	nr	nr	nr	nr	nr
Others/multiple	1 (12.5)	1 (12.5)	4 (50)	4 (26.7)	4 (26.7)	4 (30.8)	50 (21.5)	41 (18.3)	57 (24.8)	26 (33.8)	19 (24.1)	21 (27.6)	24 (32.0)	nr	nr	nr	nr
**Time on dialysis, yr, mean (SD)**	9.6 (6.7)	7.6 (4.9)	8 (5.3)	nr	nr	nr	-	-	-					nr	nr	nr	nr
**Time on dialysis, yr, median (IQR)**	-	-	-	nr	nr	nr	4.05 (2.0-7.2)	4.0 (2.0-7.5)	3.85 (1.8-7.0)	3.7 (1.9-6.3)	4.3 (1.7-7.3)	4.2 (2.0-7.5)	3.0 (1.1-6.9)	nr	nr	nr	nr
**Dialysis access, n (%)**	-	-	-	-	-	-	-	-	-	-	-	-	-	-	-	-	-
Catheter	0*	0*	0*	8 (53.3)	9 (60.0)	8 (61.5)	19 (8.2)	24 (10.7)	28 (12.2)	11 (14.3)	7 (8.9)	8 (10.5)	5 (6.7)	nr	nr	nr	nr
AV fistula	nr	nr	nr	6 (40.0)	5 (33.3)	5 (38.5)	191 (82.3)	180 (80.4)	189 (82.2)	62 (80.5)	65 (82.3)	61 (80.3)	64 (85.3)	nr	nr	nr	nr
AV graft	nr	nr	nr	1 (6.7)	1 (6.7)	0 (0)	22 (9.5)	20 (8.9)	13 (5.7)	4 (5.2)	7 (8.9)	7 (9.2)	6 (8.0)	nr	nr	nr	nr
**HD circuit anticoagulation, n (%)**	0*	0*	0*	12 (80.0)	14 (93.3)	11 (84.6)	221 (95.3)	218 (97.3)	218 (94.8)	75 (97.4)	73 (92.4)	74 (97.4)	74 (98.7)	nr	nr	nr	nr
**Comorbidities, n (%)**	-	-	-	-	-	-	-	-	-	-	-	-	-	-	-	-	-
Hypertension	8 (100)	8 (100)	8 (100)	nr	nr	nr	213 (91.8)	212 (94.6)	215 (93.5)	70 (90.9)	75 (94.9)	72 (94.7)	74 (98.7)	nr	nr	nr	nr
Diabetes	1 (12.5)	4 (50)	5 (62.5)	nr	nr	nr	90 (38.8)	82 (36.6)	87 (37.8)	27 (35.1)	27 (34.2)	26 (34.2)	27 (36.0)	nr	nr	nr	nr
Coronary heart disease	nr	nr	nr	nr	nr	nr	56 (24.1)	61 (27.2)	47 (20.4)	14 (18.2)	21 (26.6)	17 (22.4)	12 (16.0)	nr	nr	nr	nr
Myocardial infarction	nr	nr	nr	nr	nr	nr	17 (7.3)	15 (6.7)	11 (4.8)	4 (5.2)	9 (11.4)	7 (9.2)	4 (5.3)	nr	nr	nr	nr
Peripheral artery disease	nr	nr	nr	nr	nr	nr	18 (7.8)	23 (10.3)	22 (9.6)	9 (11.7)	3 (3.8)	5 (6.6)	9 (12.0)	nr	nr	nr	nr
Stroke	nr	nr	nr	nr	nr	nr	12 (5.1)	18 (7.7)	18 (7.7)	8 (10.4)	2 (2.5)	7 (9.2)	5 (6.7)	nr	nr	nr	nr
Atrial fibrillation	nr	nr	nr	nr	nr	nr	15 (6.5)	17 (7.6)	14 (6.1)	3 (3.9)	5 (6.3)	4 (5.3)	2 (2.7)	nr	nr	nr	nr
Previous cardiovascular event[a]	nr	nr	nr	nr	nr	nr	37 (15.9)	43 (19.2)	40 (17.4)	16 (20.8)	13 (16.5)	16 (21.1)	11 (14.7)	nr	nr	nr	nr
Atherosclerosis[b]	nr	nr	nr	nr	nr	nr	81 (34.9)	86 (38.4)	82 (35.7)	nr	nr	nr	nr	nr	nr	nr	nr
History of VTE	nr	nr	nr	nr	nr	nr	8 (3.4)	10 (4.5)	10 (4.3)	nr	nr	nr	nr	nr	nr	nr	nr
Cardiovascular disease[c]	2 (25)	4 (50)	2 (25)	nr	nr	nr	nr	nr	nr	nr	nr	nr	nr	nr	nr	nr	nr
History of extremity amputation	1 (12.5)	2 (25)	0 (0)	nr	nr	nr	nr	nr	nr	nr	nr	nr	nr	nr	nr	nr	nr
Cancer	0 (0)	0 (0)	1 (12.5)	nr	nr	nr	0*	0*	0*	0*	0*	0*	0*	0*	0*	0*	0*
**Low dose aspirin, n (%)**	0*	0*	0*	5 (33.3)	7 (46.7)	6 (46.2)	97 (41.8)	95 (42.4)	98 (42.6)	36 (46.8)	38 (48.1)	35 (46.1)	33 (44.0)	nr	nr	nr	nr

*In the study by Lorentz et al, patients with catheter access and patients with antiplatelet therapy were excluded, and all included patients received heparin-free dialysis sessions and. In CONVERT, RE-THINC, and EMERALD, patients with (active) cancer were excluded. [a]CONVERT and RE-THINC: previous cardiovascular events include stroke, transient ischemic attack, myocardial infarction, deep vein thrombosis and pulmonary embolism. [b]CONVERT: atherosclerosis was defined as a history of ischemic stroke, transient ischemic attack, unstable angina, myocardial infarction, peripheral artery disease or aortic aneurysm. [c]Lorentz et al: cardiovascular disease includes cardiac surgery, stent placement, coronary artery disease, aneurism, congestive heart failure, stroke, and myocardial infarction. Yr, years; BMI, body mass index; nr, not reported; SD, standard deviation; IQR, interquartile range; HD, hemodialysis; VTE, venous thromboembolism.

Table S6: Risk of bias assessment using the revised Cochrane risk of bias tool (ROB 2)

	**Lorentz et al**	**CS4**	**CONVERT**	**RE-THINC**	**EMERALD**
**Randomization process**	Low risk	Low risk	Low risk	Low risk	Low risk
**Deviations from intended interventions**	Low risk	Low risk	Some concerns	Low risk	Some concerns
**Missing outcome data**	Low risk	Low risk	Low risk	Low risk	Low risk
**Measurement of the outcome**	High risk	Some concerns	Low risk	Low risk	Low risk
**Selection of the reported result**	Some concerns	Some concerns	Low risk	Low risk	Low risk
**Overall**	High risk	Some concerns	Some concerns	Low risk	Some concerns

Table S7: Definitions of bleeding and thromboembolic events

	Lorentz et al	CS4	CONVERT	RE-THINC	EMERALD
**Clinically relevant bleeding**	Not given	Not given	Composite of major and clinically relevant non-major bleeding	Composite of major and clinically relevant non-major bleeding	Composite of major and clinically relevant non-major bleeding
**Major bleeding**	Not given	Not given	Overt bleeding and associated with a decrease in hemoglobin of 2 g/dl or more; necessitating transfusion of two or more units of blood; occurring in a critical area or organ; or contributing to death	ISTH definition [S1]: Fatal bleeding, symptomatic bleeding in a critical area or organ, such as intracranial, intraspinal, intraocular, retroperitoneal, intra-articular or pericardial, or intramuscular with compartment syndrome, and/or bleeding causing a fall in hemoglobin level of 20 g/L (1.24 mmol/L) or more, or leading to transfusion of two or more units of whole blood or red cells	Fatal bleeding; symptomatic bleeding in a critical area or organ, such as intracranial, intraspinal, intraocular, retroperitoneal, intraarticular if in a major joint, or pericardial, or intramuscular with compartment syndrome, clinically overt bleeding leading to transfusion of greater than or equal to (>=) 2 units of packed red blood cells or whole blood or a fall in hemoglobin of 20 g/L (1.24 mmol/L) or more within 24 hours
**Clinically relevant non-major bleeding**	Not given	Not given	Overt bleeding not meeting criteria for major bleeding, but necessitating medical examination or intervention, or having clinical consequences	ISTH definition [S2]: Any sign or symptom of hemorrhage (e.g., more bleeding than would be expected for a clinical circumstance, including bleeding found by imaging alone) that does not fit the criteria for the ISTH definition of major bleeding but does meet at least one of the following criteria:<br>a.) requiring medical intervention by a healthcare professional<br>b.) leading to hospitalization or increased level of care<br>c.) prompting a face to face (i.e., not just a telephone or electronic communication) evaluation	Overt bleeding not meeting the criteria for MB but that resulted, in either medical examination, intervention, or had clinical consequences for a participant
**Thromboembolic event**	Not reported	Not reported	Composite of vascular death due to myocardial infarction, stroke or pulmonary or systemic embolism; nonfatal myocardial infarction or stroke; major amputation of	Composite of fatal or nonfatal myocardial infarction, ischemic stroke, acute limb ischemia and/or major amputation, systemic	Not given

			vascular etiology; acute limb ischemia and symptomatic venous thromboembolism	embolism, and symptomatic venous thromboembolism	

References for table:

S1 Schulman S, Kearon C. Definition of major bleeding in clinical investigations of antihemostatic medicinal products in non-surgical patients. Journal of Thrombosis and Haemostasis. 2005; 3: 692-4. https://doi.org/10.1111/j.1538-7836.2005.01204.x.

S2 Kaatz S, Ahmad D, Spyropoulos AC, Schulman S. Definition of clinically relevant non-major bleeding in studies of anticoagulants in atrial fibrillation and venous thromboembolic disease in non-surgical patients: communication from the SSC of the ISTH. Journal of Thrombosis and Haemostasis. 2015; 13: 2119-26. https://doi.org/10.1111/jth.13140.

Figure S1: Forest plot for clinically relevant bleeding (CRB) events excluding the study by Lorentz et al

Study	FXI/XIa Inhibitor CRB events	FXI/XIa Inhibitor Patients (n)	Placebo CRB events	Placebo Patients (n)	Weight	Odds Ratio [95% CI]
CS4	1	30	1	13	5.1%	0.42 [0.04, 4.48]
CONVERT	27	456	18	230	75.7%	0.74 [0.40, 1.36]
RE-THINC	12	232	3	75	19.2%	1.17 [0.35, 3.96]
RE Model (Q = 0.73, df = 2, p = 0.695; I^2 = 0.0%)	**40**	**718**	**22**	**318**	**100.0%**	**0.78 [0.46, 1.33]**

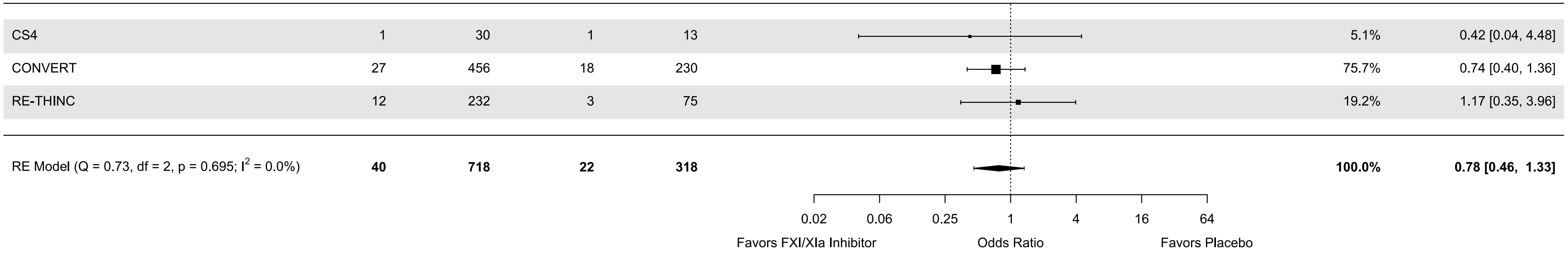

CRB, clinically relevant bleeding; CI, confidence interval; RE, random effects.

Figure S2: Forest plots for clinically relevant bleeding (CRB) and thromboembolic (TE) events including EMERALD

	FXI/XIa Inhibitor		Placebo			
**Study**	**CRB events**	**Patients (n)**	**CRB events**	**Patients (n)**	**Weight**	**Odds Ratio [95% CI]**
Lorentz et al	1	16	0	8	2.2%	1.65 [0.06, 44.97]
CS4	1	30	1	13	4.3%	0.42 [0.04, 4.48]
CONVERT	27	456	18	230	63.2%	0.74 [0.40, 1.36]
RE-THINC	12	232	3	75	16.0%	1.17 [0.35, 3.96]
EMERALD	8	157	3	53	14.3%	0.82 [0.23, 2.97]
RE Model (Q = 0.92, df = 4, p = 0.922; I^2 = 0.0%)	**49**	**891**	**25**	**379**	**100.0%**	**0.80 [0.49, 1.30]**

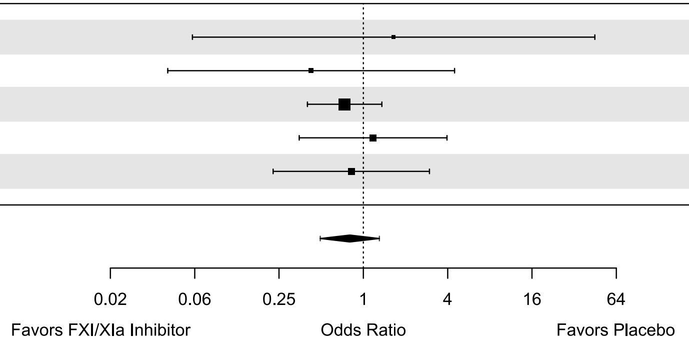

	FXI/XIa Inhibitor		Placebo			
**Study**	**TE events**	**Patients (n)**	**TE events**	**Patients (n)**	**Weight**	**Odds Ratio [95% CI]**
CONVERT	9	456	7	230	65.7%	0.63 [0.24, 1.67]
RE-THINC	3	232	1	75	16.6%	0.76 [0.11, 5.22]
EMERALD	4	157	1	53	17.6%	1.03 [0.16, 6.69]
RE Model (Q = 0.21, df = 2, p = 0.902; I^2 = 0.0%)	**16**	**845**	**9**	**358**	**100.0%**	**0.71 [0.32, 1.56]**

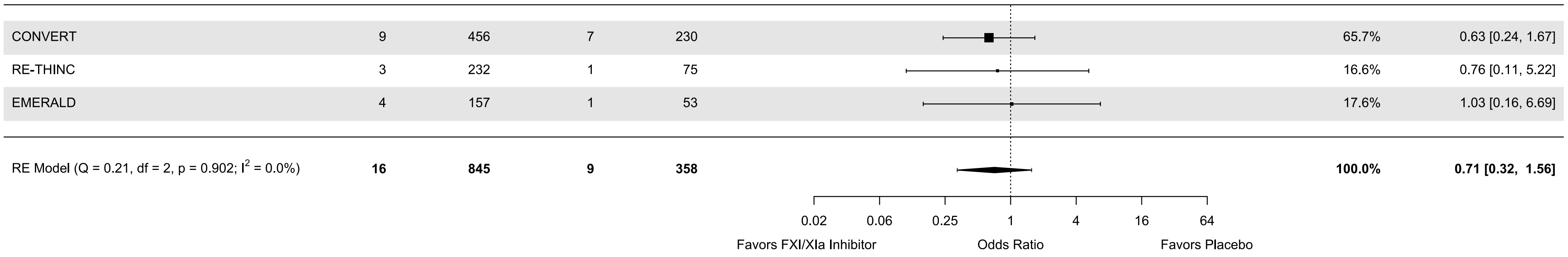

CRB, clinically relevant bleeding; CI, confidence interval; RE, random effects; TE, thromboembolic.

Figure S3: Forest plots for clinically relevant bleeding (CRB) and thromboembolic (TE) events comparing the highest dosing groups of FXI/XIa inhibitors per study to placebo

	FXI/XIa Inhibitor		Placebo			
**Study**	**CRB events**	**Patients (n)**	**CRB events**	**Patients (n)**	**Weight**	**Odds Ratio [95% CI]**
Lorentz et al	1	8	0	8	3.7%	3.40 [0.12, 96.70]
CS4	1	15	1	13	7.2%	0.86 [0.08, 9.37]
CONVERT	11	224	18	230	71.2%	0.62 [0.29, 1.32]
RE-THINC	3	76	3	75	17.9%	0.99 [0.22, 4.50]
RE Model (Q = 1.16, df = 3, p = 0.762; I^2 = 0.0%)	**16**	**323**	**22**	**326**	**100.0%**	**0.73 [0.39, 1.39]**

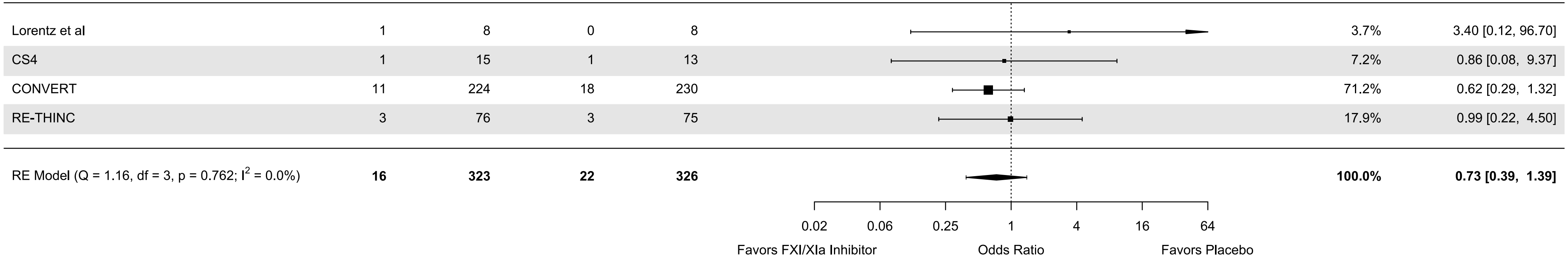

	FXI/XIa Inhibitor		Placebo			
**Study**	**TE events**	**Patients (n)**	**TE events**	**Patients (n)**	**Weight**	**Odds Ratio [95% CI]**
CONVERT	6	224	7	230	82.1%	0.89 [0.31, 2.58]
RE-THINC	1	76	1	75	17.9%	0.99 [0.10, 9.70]
RE Model (Q = 0.01, df = 1, p = 0.934; I^2 = 0.0%)	**7**	**300**	**8**	**305**	**100.0%**	**0.90 [0.34, 2.38]**

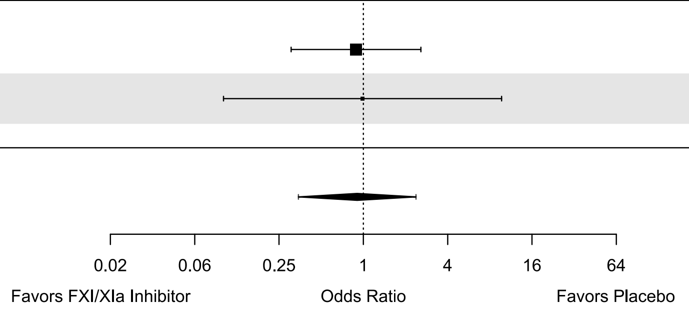

The highest dosing groups in the FXI/XI inhibitor arms were as follows: 0.5 mg/kg gruticibart in the study by Lorentz et al, 300 mg IONIS-FXI$_{Rx}$ in the CS4 study, 210 mg loading and 105 mg maintenance osocimab in the CONVERT study, and 120 mg fesomersen in the RE-THINC study. CRB, clinically relevant bleeding; CI, confidence interval; RE, random effects; TE, thromboembolic.

Figure S4: Forest plots for major bleeding (MB) and clinically relevant non-major bleeding (CRNMB) events excluding the study by Lorentz et al

	FXI/XIa Inhibitor		Placebo			
**Study**	**MB events**	**Patients (n)**	**MB events**	**Patients (n)**	**Weight**	**Odds Ratio [95% CI]**
CS4	1	30	1	13	16.3%	0.42 [0.04, 4.48]
CONVERT	5	456	7	230	73.2%	0.36 [0.12, 1.10]
RE-THINC	4	232	0	75	10.5%	2.97 [0.16, 55.88]
RE Model (Q = 1.73, df = 2, p = 0.420; I^2 = 0.0%)	**10**	**718**	**8**	**318**	**100.0%**	**0.46 [0.18, 1.20]**

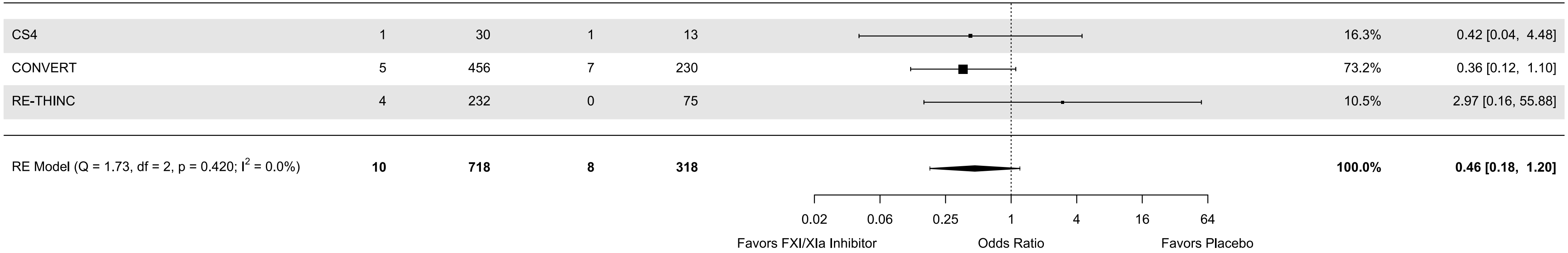

	FXI/XIa Inhibitor		Placebo			
**Study**	**CRNMB events**	**Patients (n)**	**CRNMB events**	**Patients (n)**	**Weight**	**Odds Ratio [95% CI]**
CS4	0	30	0	13	2.5%	0.44 [0.01, 23.50]
CONVERT	22	456	11	230	73.4%	0.99 [0.48, 2.05]
RE-THINC	8	232	3	75	24.1%	0.78 [0.22, 2.80]
RE Model (Q = 0.23, df = 2, p = 0.892; I^2 = 0.0%)	**30**	**718**	**14**	**318**	**100.0%**	**0.92 [0.49, 1.71]**

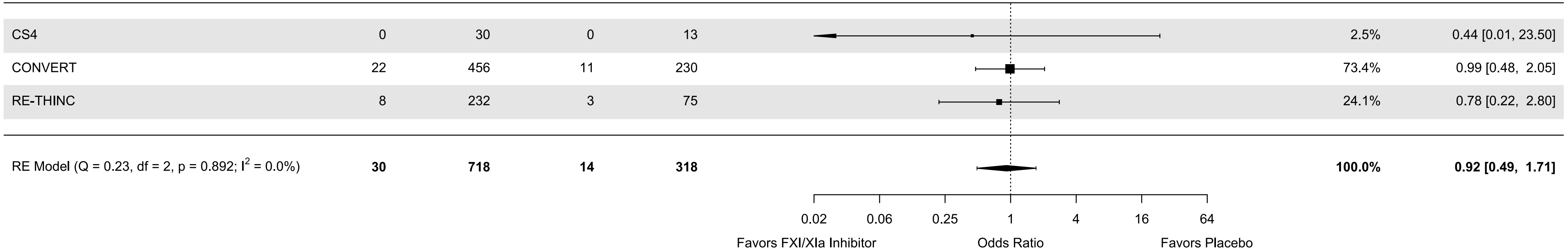

MB, major bleeding; CRNMB, clinically relevant non-major bleeding; CI, confidence interval; RE, random effects.

Figure S5: Forest plot for all-cause mortality excluding the study by Lorentz et al

Study	FXI/XIa Inhibitor Deaths	FXI/XIa Inhibitor Patients (n)	Placebo Deaths	Placebo Patients (n)	Weight	Odds Ratio [95% CI]
CS4	1	30	1	13	18.4%	0.42 [0.04, 4.48]
CONVERT	21	456	11	230	47.4%	0.94 [0.45, 1.96]
RE-THINC	3	232	6	75	34.2%	0.16 [0.04, 0.61]
**RE Model (Q = 5.24, df = 2, p = 0.073; I^2 = 60.1%)**	**25**	**718**	**18**	**318**	**100.0%**	**0.45 [0.13, 1.52]**

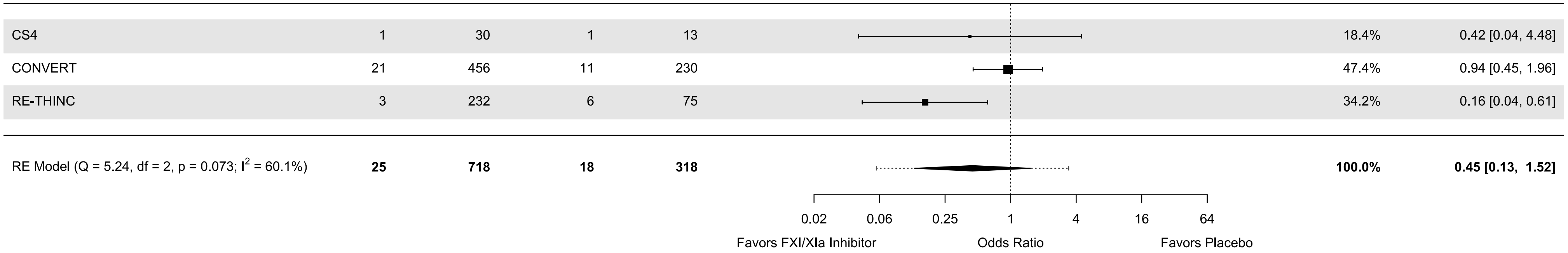

CI, confidence interval; RE, random effects.

Figure S6: Forest plot for all-cause mortality including EMERALD

Study	FXI/XIa Inhibitor Deaths	FXI/XIa Inhibitor Patients (n)	Placebo Deaths	Placebo Patients (n)	Weight	Odds Ratio [95% CI]
Lorentz et al	0	16	0	8	4.0%	0.52 [0.01, 28.30]
CS4	1	30	1	13	10.2%	0.42 [0.04, 4.48]
CONVERT	21	456	11	230	39.4%	0.94 [0.45, 1.96]
RE-THINC	3	232	6	75	23.3%	0.16 [0.04, 0.61]
EMERALD	6	157	3	53	23.1%	0.62 [0.16, 2.36]
RE Model (Q = 5.24, df = 4, p = 0.263; I^2 = 37.3%)	**31**	**891**	**21**	**379**	**100.0%**	**0.51 [0.22, 1.18]**

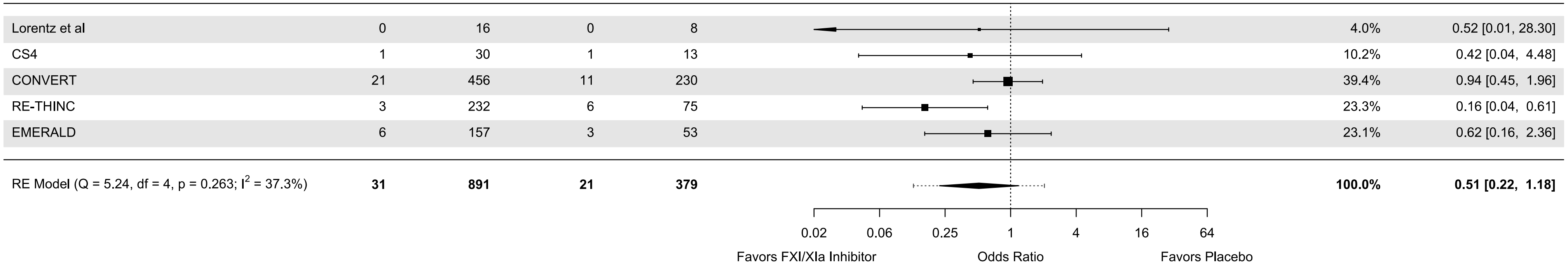

CI, confidence interval; RE, random effects.

Figure S7: Forest plots for major bleeding (MB) and clinically relevant non-major bleeding (CRNMB) events comparing the highest dosing groups of FXI/XIa inhibitors per study to placebo

Study	FXI/XIa Inhibitor MB events	FXI/XIa Inhibitor Patients (n)	Placebo MB events	Placebo Patients (n)	Weight	Odds Ratio [95% CI]
Lorentz et al	1	8	0	8	13.8%	3.40 [0.12, 96.70]
CS4	1	15	1	13	23.9%	0.86 [0.08, 9.37]
CONVERT	2	224	7	230	46.3%	0.33 [0.08, 1.42]
RE-THINC	2	76	0	75	16.1%	5.07 [0.24, 107.34]
RE Model (Q = 3.50, df = 3, p = 0.321; I^2 = 23.7%)	**6**	**323**	**8**	**326**	**100.0%**	**0.89 [0.23, 3.40]**

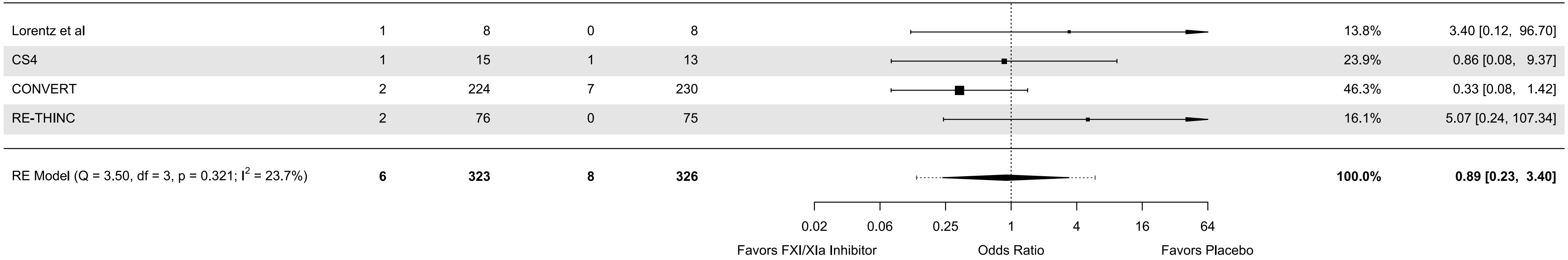

Study	FXI/XIa Inhibitor CRNMB events	FXI/XIa Inhibitor Patients (n)	Placebo CRNMB events	Placebo Patients (n)	Weight	Odds Ratio [95% CI]
Lorentz et al	0	8	0	8	3.7%	1.00 [0.02, 56.46]
CS4	0	15	0	13	3.7%	0.87 [0.02, 46.95]
CONVERT	9	224	11	230	76.8%	0.84 [0.35, 2.03]
RE-THINC	1	76	3	75	15.8%	0.41 [0.06, 2.86]
RE Model (Q = 0.46, df = 3, p = 0.928; I^2 = 0.0%)	**10**	**323**	**14**	**326**	**100.0%**	**0.76 [0.35, 1.64]**

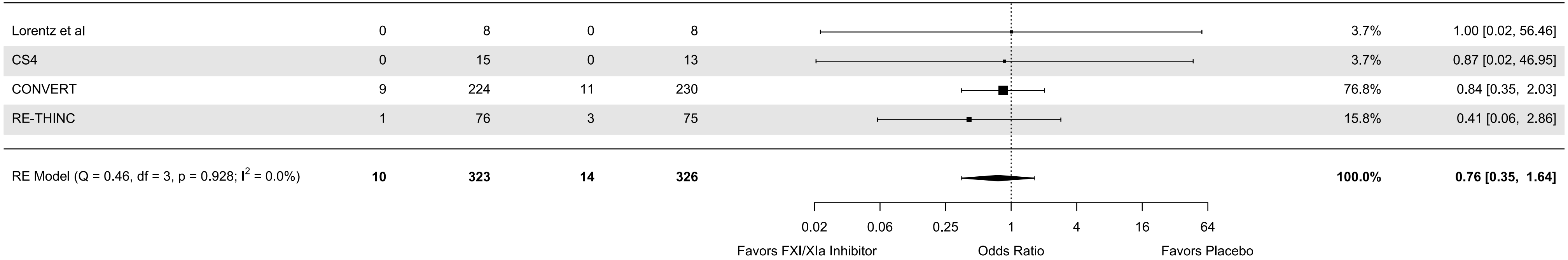

The highest dosing groups in the FXI/XI inhibitor arms were as follows: 0.5 mg/kg gruticibart in the study by Lorentz et al, 300 mg IONIS-FXI$_{Rx}$ in the CS4 study, 210 mg loading and 105 mg maintenance osocimab in the CONVERT study, and 120 mg fesomersen in the RE-THINC study. MB, major bleeding; CRNMB, clinically relevant non-major bleeding; CI, confidence interval; RE, random effects.

Figure S8: Forest plot for all-cause mortality comparing the highest dosing groups of FXI/XIa inhibitors per study to placebo

	FXI/XIa Inhibitor		Placebo			
**Study**	**Deaths**	**Patients (n)**	**Deaths**	**Patients (n)**	**Weight**	**Odds Ratio [95% CI]**
Lorentz et al	0	8	0	8	5.1%	1.00 [0.02, 56.46]
CS4	0	15	1	13	7.6%	0.27 [0.01, 7.19]
CONVERT	9	224	11	230	64.7%	0.84 [0.35, 2.03]
RE-THINC	1	76	6	75	22.6%	0.21 [0.03, 1.29]
RE Model (Q = 2.12, df = 3, p = 0.548; I^2 = 12.1%)	**10**	**323**	**18**	**326**	**100.0%**	**0.57 [0.23, 1.44]**

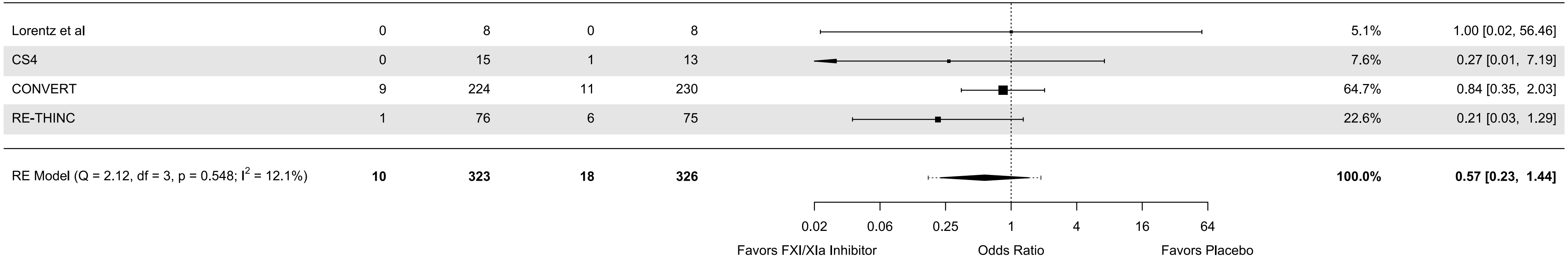

The highest dosing groups in the FXI/XI inhibitor arms were as follows: 0.5 mg/kg gruticibart in the study by Lorentz et al, 300 mg IONIS-FXI$_{Rx}$ in the CS4 study, 210 mg loading and 105 mg maintenance osocimab in the CONVERT study, and 120 mg fesomersen in the RE-THINC study. CI, confidence interval; RE, random effects.
